# GC-MS based nutritional and aroma profiling of date palm seeds collected from different Egyptian cultivars for valorization purposes

**DOI:** 10.1038/s41598-025-00171-7

**Published:** 2025-05-13

**Authors:** Walaa M. Ismail, Ahmed Zayed, Nehal S. Ramadan, Sarah T. Sakna, Mohamed A. Farag

**Affiliations:** 1 Pharmacognosy Department, College of Pharmacy, Cairo University, Kasr El Aini St, P.B. 11562, Cairo, Egypt; 2 Pharmacognosy Department, College of Pharmacy, Tanta University, Elguish Street (Medical Campus), Tanta, 31527 Egypt; 3 Chemistry of Tanning Materials and Leather Technology Department, National Research Centre, Dokki, 12622 Cairo Egypt; 4 Faculty of Healthcare, Saxony Egypt University (SEU), Badr city, Egypt; 5 College of Pharmacy, Department of Pharmacognosy, Cairo University, Cairo, Egypt

**Keywords:** Chemometrics, Date palm seed, GC-MS, HS-SPME, Metabolomics, *Phoenix dactylifera* L, Metabolomics, Chemical tools

## Abstract

Date palm (*Phoenix dactylifera* L.) is a globally edible fruit and a traditional dietary component in various cultures. The fruit’s fleshy part is consumed for its nutritional value, while the seeds are discarded or valorized for oil production and as a coffee substitute. The current study aimed to investigate date seeds’ metabolome, in addition to macro- and micro-elements composition within12 major Egyptian cultivars (cvs.) for the first time using gas chromatography coupled with mass spectrometry (GC-MS). Post-silylation GC-MS analysis and headspace coupled with solid-phase microextraction (HS-SPME) were used for nutrients and aroma profiling in roasted seeds, respectively. Furthermore, multivariate data analyses were employed for samples classification and markers identification *via* principal component analysis (PCA) and orthogonal projection to least square discriminant analysis (OPLS-DA). Models are further validated by permutation test. Moreover, absolute quantification of potential markers was attempted based on reference standards A total of 101 and 65 nutrient and aroma metabolites were annotated, respectively. Fatty acids/esters (38 peaks), sugars (18), organic acids (17), sugar alcohols (7), steroids/triterpenoids (5), alcohols and aldehydes (6), in addition to flavonoids (1) and phenolic acids (3) were identified as major components in GC-MS post-silylation platform. ‘’Khalas’’ cv. seed appeared the most nutritive being enriched in sugars and fatty acids/esters. Moreover, date seed volatiles from different cvs. were dominated by fatty acids/esters (19 peaks), esters (6), and phenols/ethers (9). Anethole (peak 47) was the most abundant at 9.1–23.3% of seeds contributing to their unique aroma, especially ‘’Barhi’’ a premium date cv. PCA score plot of primary metabolites’ dataset revealed for 1-monopalmitin and monostearin as potential markers for ‘’Aref’’ and ‘’Khalas’’. Furthermore, ‘’Barhi’’, ‘’Omeldehn’’, and ‘’Lolo’’ cvs. showed comparable aroma profile and in partial agreement with nutrient results. OPLS-DA model revealed that anethole, estragole, methyl esters of dodecanoic acid and octanoic acid were characteristic in case of ‘’Barhi’’ cv. which are likely to impart a fine aroma and flavor. With regards to minerals, ‘’Zamli’’, ‘’Barhi’’, and ‘’Hasawi’’ cvs. were most rich in calcium, copper, and selenium, respectively. This study offers new perspectives for the phytochemical makeup and valorization potentials of date palm seeds. Fatty acids/esters and sugars were the major components in date palm seeds found enriched in ‘’Khalas’’ cv, while anethole, estragole, methyl esters of dodecanoic acid and octanoic acid were potential markers of ‘’Barhi’’ cultivar. Such extensive profiling identified premium cvs. to be considered for food applications.

**Supplementary Information:**

The online version contains supplementary material available at 10.1038/s41598-025-00171-7.

## Introduction

Date palm (*Phoenix dactylifera* L.) is one of the oldest widely used crop trees known worldwide, extensively grown and consumed as a fundamental dietary component for its high nutritive value in the Middle East especially in Southern Asia and North Africa^1^ with more than 70% of the world’s dates cultivated in countries in the Middle East and Northern Africa^2^. Particularly, Egypt has grown at a rate of 222% between 1990 and 2021, making it the world’s largest date producer^2–4^. Recently, about 79 cultivars (cvs.) are cultivated in different regions all over Egypt^5^. ‘’Zaghloul’’ and ‘’Hasawi’’ are the most common cvs., whereas ‘’Khalas’’, ‘’Barhi’’, and ‘’Omeldehn’’ are exotic types that became widely cultivated in Egypt^5^.

Date palm fruits exert these biological impacts attributed to several classes *viz.* flavonoids, phenolic acids, hydroxycinnamates, carotenoids, sugars, sterols, and fatty acids^4,6–8^. However, date fruit processing industries create several by-products, including entire cull dates, leaves, pollen, and date seeds. Due to adverse effect on the environment^9^, they are used as animal feed for their valuable constituents including, dietary fibers, sugars, fatty acids, and flavonoids^1,6^.

Date palm seeds represent a good source of oil (5–13%), which is rich in phenolics, tocopherols, and phytosterols^10^. Date palm seeds have recently become a point of interest in phytochemical studies to assess their phytoconstituents and pharmacological effects, and to amount for *ca.* 15% of the weight of the fresh date fruit^11^. In folk medicine, seeds of date palm are widely used for the management of several diseases, including liver disorders, diabetes, and gastrointestinal complaints^3^. Several bioassays on date seeds proved their antimicrobial, antioxidant, cytotoxic, antidiabetic and anti-inflammatory activities^11^. The aqueous extract of date palm seeds was reported to exert a hepatoprotective effect in rats^3^. These effects warranted for more studies on date seeds in an attempt for valorization purposes other than being used as animal feed^12,13^ or in humans as a coffee substitute post roasting to improve their sensory properties^14^.

The metabolite composition of roasted and unroasted seeds of ‘’Majdool’’ cv. seed as coffee substitute was assessed using headspace-solid phase microextraction coupled with gas chromatography–mass spectrometry (HS-SPME/GC–MS) in addition to GC–MS for silylated components^15^, revealing that roasting affected date composition especially aroma compounds. Targeting more secondary non-volatile metabolites, seeds were explored *via* UPLC–MS and NMR, with 67 metabolites identified belonging to phenolic acids, flavonols, fatty acids, sphingolipids, steroids, and saponins^6^, and adding to date seed functional properties. Additionally, 14 cvs. of date seeds from Saudi Arabia were tested for their fatty acids composition by GC-MS, with oleic acid found as major component among the majority of the investigated cvs^16^. Oleic acid was also identified as the major fatty acid in the seed oils of two Tunisian cvs., while lauric acid and palmitic acid were the main saturated fatty acids in ‘’Deglet Nour’’ and ‘’Allig’’ cvs., respectively^17^, and suggestive for difference in fatty acids composition among date seeds which has yet to be revealed as analysed using omics technologies.

Recent studies have investigated the various phytoconstituents and the importance of the Egyptian cvs. e.g. anti-inflammatory activity of ‘’Sewy’’ cv^18^, antimicrobial and antiviral properties of Tamr El Wadi cv^19^. Although the phytochemical profile of date palm seeds and seed oil were previously studied^20,21^, it was still of interest to assess the difference in volatile or non-volatile metabolites diversity among several cvs. in Egypt; the chief date producer worldwide^2–4^, to assist their characterization using untargeted metabolites profiling and discrimination using chemometric tools^22^. The approach of using GC-MS is most suited for aroma profiling being most sensitive allowing excellent resolve of complex volatile blend further post silylation. It is also suited for monitoring for low molecular weight primary metabolites and has been extensively reported in similar type of work^15^. In addition, phytochemical analysis of volatile organic compounds (VOCs) has not been investigated for date palm cvs. using SPME/GC-MS, which is routinely applied for exploring aroma composition in food and herbal products^23^, and should aid in identifying cvs. with the best aroma profile to be used as coffee substitute. In the Middle East, roasted dates are commonly used as coffee substitute commercially owing to their comparable sensory attributes^15^.

Considering the complexity of metabolites datasets, chemometric analysis is typically employed for analyzing data sets generated from MS-based analysis and assessment variation between different samples using unsupervised principal component analysis (PCA) and supervised orthogonal projection to least squares discriminant analysis (OPLS-DA). In recent years, dealing with huge data sets arising from wide range of cvs. accomplished the importance of applying metabolomics in analysis to accurately characterize and distinguish different cvs. in aspect to their metabolite profiles, which assist providing clear discrimination of different cvs. to be used in choosing the most reliable beneficial candidates based on the phytochemical profile^24,25^.

Minerals are either macronutrients (e.g., calcium (Ca), magnesium (Mg), and phosphorus (P) pr micronutrients, (e.g., iron (Fe), selenium (Se), copper (Cu), manganese (Mn) and boron (B), likewise important for adequate functions of the body especially bones, muscles and nerves^26,27^, in addition to their inclusion in enzymes and body hormones^28^. In Saudi palm seeds, Ca, Mg, and Fe were reported as major forms^29^. Although some Egyptian date palm seeds were evaluated for their mineral content (e.g. ‘’Sukari’’^30^, ‘’Khalas’’^31^, and ‘’Barhi’’^32^ cvs.), exploration of the mineral content of more cvs. yet to be examined in this study. Searching for natural available sources of minerals has been an influential leading target for numerous research^26,27,33,34^.

Hence, the main goal of this study was to evaluate diversity in primary, secondary, and mineral profiles in date seeds represented by 12 cvs. grown in Egypt using multiplex metabolomics approach to assess profiling of date waste products. To guide cvs. classification and identification of markers for each cv., chemometric tools exemplified by unsupervised multivariate data analyses (e.g., principal component analysis (PCA) and hierarchical clustering analysis (HCA)), in addition to supervised methods (e.g., orthogonal projection to least squares discriminant analysis (OPLS-DA)) were employed for analysis of date palm seed metabolite profile datasets. The study also aimed to explore phytochemical profile of agricultural wastes which can contribute to sustainability in the food industry and health applications.

## Materials and methods

### Plant materials and preparation for analysis

The date palm fruits were harvested in October 2019 at the “Rutab” stage from the date orchard at Saff Agricultural Station, Giza Governorate, Egypt. The various investigated samples in this study included 12 main cvs. named following their local Arabic names; ‘Barhi’’, ‘’Omeldehn’’, ‘’Rothana’’, ‘’Lolo’’, ‘’Nabout Seif’’, ‘’Zamli’’, ‘’Khalas’’, ‘’Farsi’’, ‘’Hasawi’’, ‘’Zaghloul’’, ‘’Aref’’, and ‘’Breem’’. The palm trees were grown in Giza (Coordinates: 29.9870°N 31.2118°E) with an average of 310 monthly h of sunshine per year, temperatures ranging from 20 °C to 30 °C, and a 60% humidity level^7^. Three biological replicates of 10 dates were chosen (free of flaws and color variances). Afterward, the dates fruit were pitted manually to separate the edible part obtaining the seeds. Before storage in tight glass containers at -20 °C, the seeds were lyophilized overnight at -35 °C using a Benchtop Freeze Dryer, LYO60B-1PT, until completely dried, then pulverized with a Kenwood KHH326BK MultiOne Mixer 1000 W. Roasting was carried out for 100 mg seeds in oven for 3 h at 120 °C then the roasted seeds were ground into fine powder^15^.

The Pharmacognosy department, Faculty of Pharmacy, Cairo University (Giza, Egypt) keeps voucher specimens of fruits and seeds analyzed in this study. The sample codes used in the current study were applied after the addition of the department’s initials and year of collection. For instance, ‘’Khalas’’ was kept under the voucher code PG_CU_Khalas_2019. In addition, all experimental procedures were carried out in accordance with the relevant laws and guidelines, including the appropriate permissions for the collection of plant specimens.

### GC-MS analysis and metabolites profiling of date palm seeds

#### Primary metabolites profiling using GC-MS post silylation

The primary metabolites of date palm seeds were analysed using GC-MS following silylation as previously described by Farag et al.^15,35^ with few modifications. Briefly, finely powdered date seeds (100 mg) were extracted with 5 mL 100% methanol aided by sonication and for 30 min at room temperature using Branson CPX-952-518R (Branson Ultrasonics, Carouge, SA Switzerland). The extracts were then centrifuged (LC-04 C 80-2 C regen lab centrifuge, Zhejiang, China) at 12,000× *g* for 10 min. Three independent replicates (*n* = 3) were done for each sample.

Then, 100 µL of the methanol extract was evaporated to dryness in opened screw-cap vials by using stream of nitrogen gas. For derivatization step, 150 µL of *N*-methyl-*N*-(trimethylsilyl)-trifluoroacetamide (MSTFA) previously mixed (1:1) with anhydrous pyridine was added to the dried methanol extract and the mixture was incubated (Yamato Scientific DGS400 Oven, QTE TECHNOLOGIES, Hanoi, Vietnam) for 45 min at 60 °C. Silylated derivatives were separated on a Rtx-5MS Restek, Bellefonte, PA, USA (30-m length, 0.25-mm inner diameter and 0.25-m film). Soluble sugars, amino acids, organic acids, and fatty acids were measured using standard curves of glucose, glycine, lactic acid, and oleic acid, and the results were represented in mg/g. The standard curves were established by preparing four serial dilutions ranging from 10 to 600 µg/mL. Calibration curves for glucose, glycine, citric acid, and stearic acid showed a correlation coefficient (R^2^ of 0.99. In addition, the detected metabolite levels were normalized to the level of the spiked internal standard, xylitol, which could be detected at 10 µg/mL.

#### Volatiles profiling of date palm seeds using HS-SPME coupled with GC-MS

Following seeds roasting, HS-SPME coupled with GC-MS was used with some modifications^36^. Briefly, 20 mg of finely pulverized seeds were placed in SPME screw-cap vials quickly sealed and set on a tray that was kept at a constant 50 °C for 30 min. During that time, SPME fibers of stable flex coated with divinylbenzene/carboxen/polydimethylsiloxane (DVB/CAR/PDMS) 1 cm long of 50/30 µm purchased from Supelco^®^ (Oakville, ON, Canada) were used for sampling by incubation into the headspace above the sample. As a control, a system blank devoid of any plant material was operated. The analysis of volatile compounds was carried out on a Schimadzu^®^ GC-17 A gas chromatograph equipped with DB-5 column (30 m x 0.25 mm i.d. x 0.25 μm film thickness; Supelco) and coupled to Schimadzu^®^ QP5050A mass spectrometer following conditions described in Refs.^6,15^. The method validation was done as previously adopted^37^.

#### Identification of non-volatile silylated and aroma compounds

Peaks were first deconvoluted using AMDIS software (www.amdis.net) before being matched to spectral data. Furthermore, AMDIS (www.amdis.net) was used to extract data abundance, with the relative content of each metabolite determined by area normalization of all responses associated to identified hits, and the average response per injection replicate calculated for each metabolite separately. Metabolites that responded in only one replicate were excluded from the average calculation. Metabolites were identified by comparing their retention indices (RI) to reference *n*-alkanes (C_11_-C_28_), mass spectral matching to NIST in addition to WILEY library database and comparison with standards when available. Peak abundance data were exported fo r multivariate data analysis by extraction using MS dial software (http://prime.psc.riken.jp/compms/msdial/main.html) previously set at parameters, including mass range (0-500 Da), MS1 tolerance for alignment (0.015 Da), retention time (0–30 min), sigma (0.7), minimum peak height (1000), and accurate mass tolerance (MS) 0.01 Da^22^.

### Multivariate data analyses and statistical analyses

Both GC-MS for primary non-volatile and volatile metabolites datasets were subjected to unsupervised multivariate data analysis (e.g., PCA, HCA) and supervised OPLS-DA using SIMCA-P version 14.1 software package (Umetrics, Umeå, Sweden). All variables were mean-centered and scaled to Pareto variance. Unsupervised PCA was used to obtain a segregation pattern of the variance of metabolites among the various seeds’ cvs. while the supervised OPLS-DA was used to verify PCA results, identify markers and obtain detailed information on the distinctions among the studied specimens. The Chemometric models with several permutations were applied for models’ validation based on the main 2 indices, including R^2^ and Q^2^. Model predictability was indicated by Q^2^, and model goodness of fit was specified by R^2^. In addition, Hotelling’s T2 was used to identify strong outliers for the OPLS-DA plot, whereas DModx (distance to the model) identifies outliers^38^.

### Mineral content in seeds of different date palm Cvs

The minerals (Ca, Mg, P, Fe, Cu, Se, Mn, and B) were determined using inductively coupled plasma atomic emission spectrometry (Thermo iCAP 6500 Duo ICP-AES Spectrometer) according to^39^. In brief, samples of 0.5 g of powdered seeds were separately weighed in a tetrafluoromethoxy (TFM) vessel. Then, a mixture of 7 mL of 65% HNO_3_ and 1 mL 10% H_2_O_2_ was added cautiously to digest the sample. The vessel was then introduced into a Milestone ETHOS 900 Microwave Lab-Station (with Easy control software) using segmented rotor (HPR 1000/10s). The digestion of samples was carried out at 1800 W for 15 min, where the temperature gradually increased to 200 °C, and then the same power was kept so the temperature continued at 200 °C for another 15 min. The samples were left to cool and deionized water was added to give final volume of 50 mL. Furthermore, significance analysis was conducted using one-way analysis of variance (ANOVA) at *p* < 0.05. The data were subsequently categorized using post-hoc Tukey analysis (*n* = 3) using Minitab^®^ statistical software version 17.1 (Minitab, LLC., USA).

## Results and discussion

### Date seeds primary metabolites profiling post-silylation

The diversity of metabolites in date palm seeds among 12 major Egyptian date cvs. was comparatively investigated *via* GC-MS technique post-silylation followed by multivariate data analyses for the first time. A total of 101 peaks were identified in seeds of different cvs. belonging to fatty acids/esters (38 peaks), sugars (18), organic acids (17), sugar alcohols (7), steroids/triterpenoids (5), alcohols, and aldehydes (6) which are expressed as mg/g, Table S1, in addition to flavonoids (1) and phenolic acids (3) represented as a relative percentile (%) in Table S2. Compared to previous GC-MS of silylated products.

**Fig. 1:**
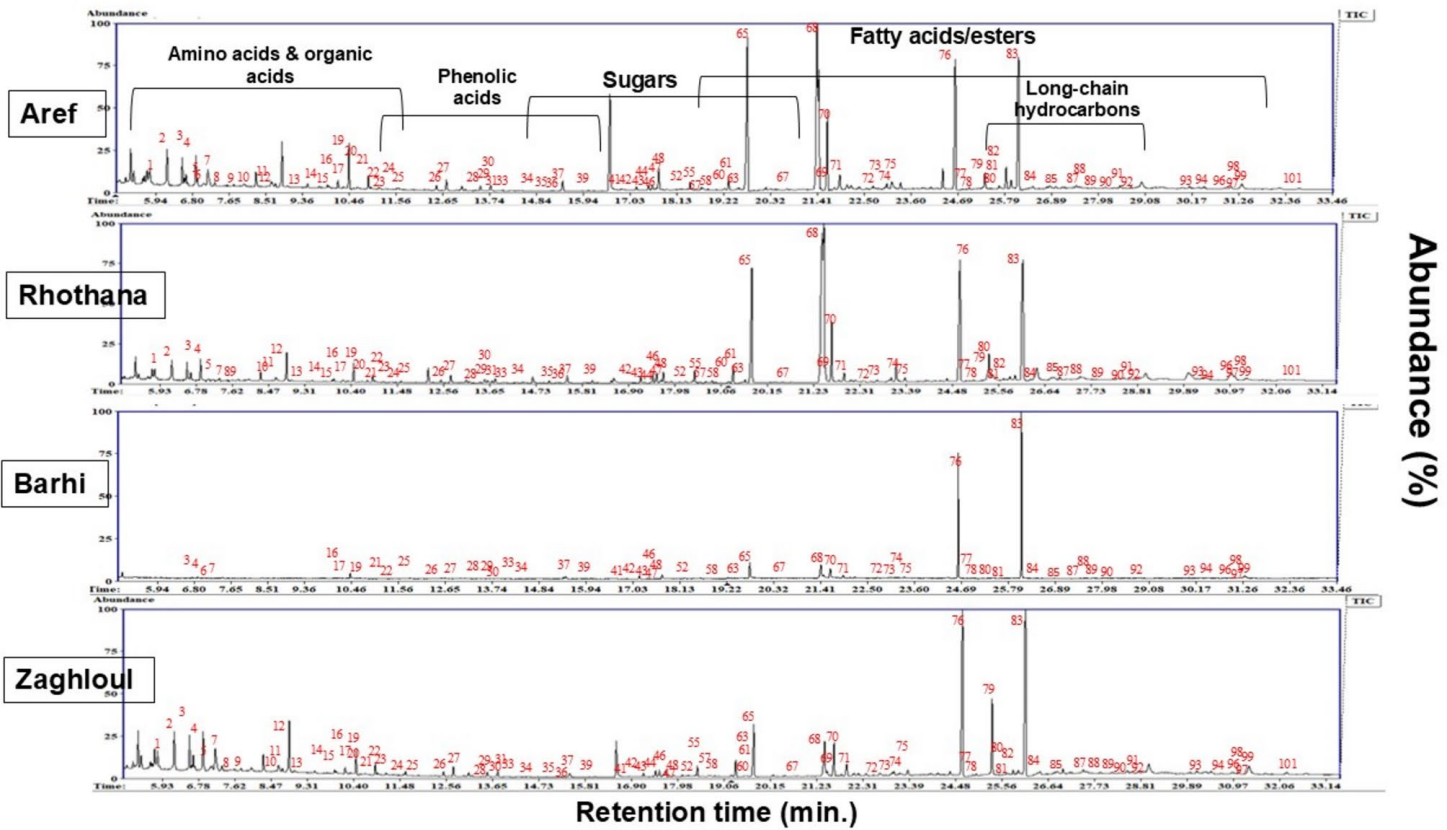
Representative GC-MS chromatograms of silylated primary metabolites in seeds of date palm (*P. dactylifera* L.) cultivars. The peak numbers denote for the identified metabolites listed in Table S1.

**Fig. 2:**
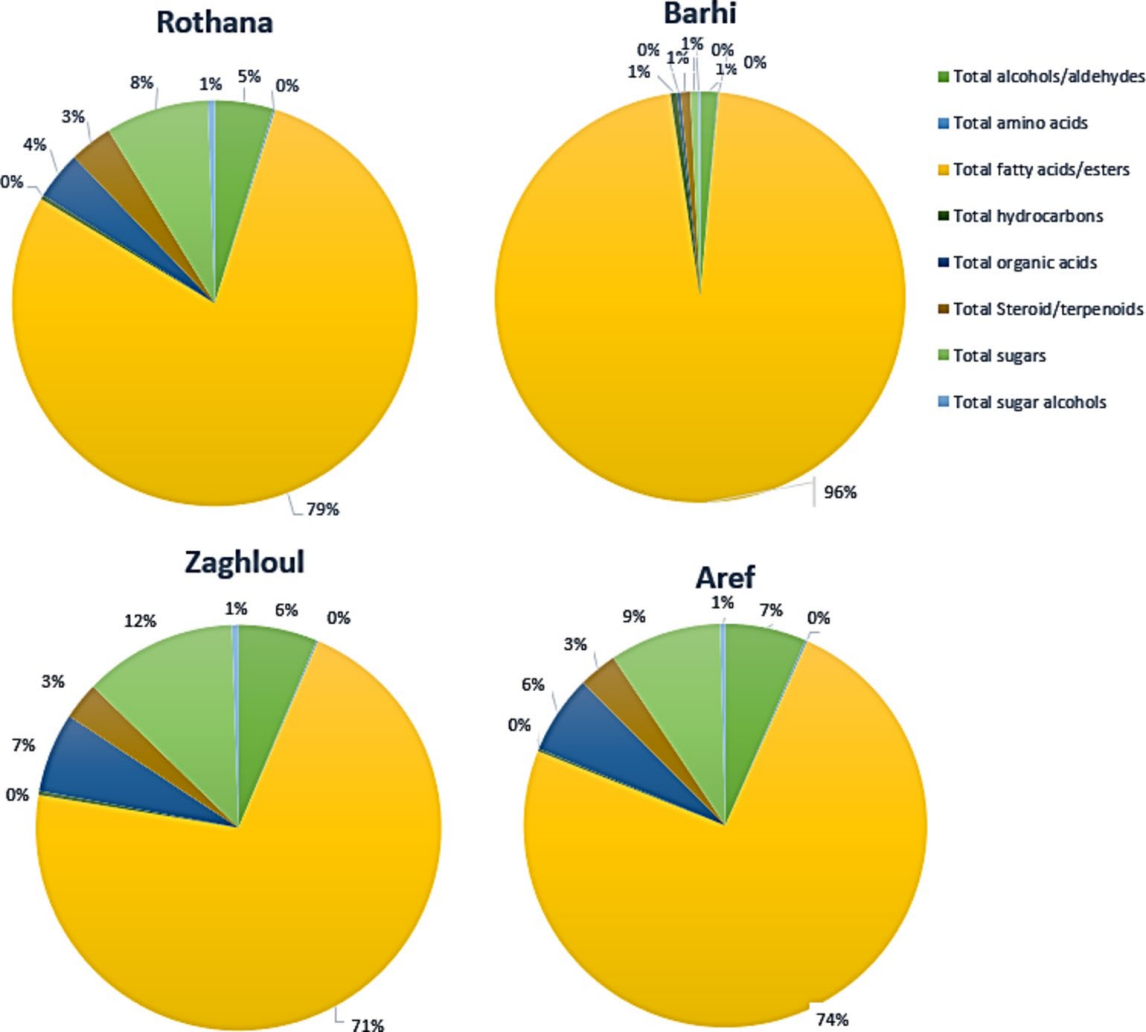
Pie chart showing the relative percentile (%) of the major classes of the identified primary metabolites derived from some representative date palm (*P. dactylifera* L.) seed cultivars. The percentile of each phytochemical class is listed in details in Table S1.

Representative GC-MS chromatograms of date palm seeds are shown in Fig. 1, *i.e.*, ‘’Aref’’, ‘’Rothana’’, ‘’Barhi’’, and ‘’Zaghloul’’. Peaks were labeled for easy identification throughout the manuscript and are consistent with the peak number assignments made in Table S1. In addition, the relative percentile of the major classes of the identified metabolites in date palm seeds of the investigated cvs. is depicted as a pie chart in Fig. 2. Fatty acids/esters represented the most abundant class, amounting to 96% of the total constituents in ‘’Barhi’’. Sugars were the second most abundant class, accounting for 12% in ‘’Zaghloul’’ cv. The presence of high abundance of sugars aids in the palatable taste of date palm seeds containing products^10,11^. The following subsections dissect the various classes of metabolites identified by GC-MS after silylation.

#### Fatty acids/esters

Fatty acids and their esters represented the major class in all cvs. denoted by 38 peaks with total amounts ranging from the least content in ‘’Farsi’’ to the highest fatty acid content in ‘’Khalas’’ seeds. ‘’Aref’’, ‘’Rothana’’, and ‘’Zaghloul’’ cvs. Free fatty acids were represented by 23 peaks which varied from medium-chain fatty acids (MCFAs), e.g., caproic acid (peak 7) and lauric acid (peak 37) to long-chain fatty acids, e.g., myristic (peak 48), and hexacosanoic acids (peak 89), Table S1.

Date palm seeds are one of the natural sources of medium-chain fatty acids, mainly caproic and lauric acids^40^. Nine medium-chain fatty acids were detected in the different cvs. represented by peaks 7, 12, 16, 25, 27, 29, 37, 39, and 42. Caproic acid (peak 7) was the major medium-chain fatty acid detected in ‘’Aref’’ and ‘’Zaghloul’’ date cvs. Lauric acid (peak 37) was found at highest level in ‘’Aref’’ (7.3 mg/g) followed by ‘’Breem’’, ‘’Rothana’’, and ‘’Zaghloul’’ cvs. at 4.7, 4.5, and 4.4 mg/g, respectively. Generally, medium-chain fatty acids are well recognized for their role in regulating fat and glucose metabolism by increasing insulin secretion^41^. Consequently, these cvs. enriched in medium-chain fatty acids such as caproic acid (peak 7) and lauric acid (peak 37) that can be considered as adjunct treatment of metabolic disorders such as diabetes, hypercholesterolemia, hypertriglyceridemia, and obesity^41^.

In addition, different long-chain fatty acids were detected in most seed cvs. exemplified by palmitic acid as the major fatty acid reaching up to 77.99 mg/g in ‘’Aref’’ and 53.0 mg/g in ‘’Rothana’’ date seeds. Regarding saturated fatty acids, stearic acid (peak 70) was detected ranging between 0.1 mg/g in ‘’Farsi’’ to 28.4 mg/g in ‘’Aref’’ cv. Stearic acid-rich foods have been reported to lower the well-known risk factor for coronary heart disease (CHD), i.e., low-density lipoprotein (LDL)-cholesterol^42^.

Unsaturated fatty acids were represented by peaks 63, 68, 69, 74, and 77, including monounsaturated omega-9 fatty acids, *i.e.*, oleic acid (peak 69), and polyunsaturated fatty acids, *i.e.*, 9-hexadecenoic (peak 63), eicosadienoic (peak 74), docosadienoic (peak 77), and linoleic acid (peak 68) (omega-6). Linoleic acid is an essential fatty acid and was the second most abundant fatty acid detected in several cvs. (e.g., 46.5 mg/g in ‘’Rothana’’ and 35.3 mg/g in ‘’Aref’’ date palm seeds). This data differed from previous reports on seeds from Saudi cvs. where oleic acid was the major fatty acid detected^16^ suggestive of geographical origin or agricultural impact on lipids profile in date seed. Comparison with previous reports showed that ‘’Rothana’’ seeds from Saudi Arabia were rich in caproic, lauric, stearic, myristic, and oleic acids consistent with the present results^16^. Other cvs. revealed from this study to be enriched in fatty acids included ‘’Aref’’ that comprised the highest levels of several key fatty acids, including, caproic (16.16 mg/g), stearic (28.43 mg/g), and myristic acids (8.21 mg/g).

Regarding fatty acid esters, 15 metabolites were annotated which mainly included monoglyceryl esters. The most abundant ester was monostearin (peak 83) ranging from high abundance in ‘’Khalas’’ (220.1 mg/g), ‘’Lolo’’ (214.2 mg/g) and ‘’Breem’’ (173.4 mg/g) date seeds to its least level in ‘’Farsi’’ (9.5 mg/g) posing ‘’Khalas’’ and ‘’Lolo’’ as valuable source of that food additive. Monostearin is first time to be detected in date seeds, and has potential application food industry as an emulsifying agent and surfactant in addition to increasing thickness to food products^43^. Monoglyceryl esters are rich in some oils, e.g., soybean (720 mg/g) and olive (260 mg/g) oils but mainly synthesized in large-scale production^44^. Glyceryl esters of unsaturated fatty acids including oleic and linoleic acids were also detected in ‘’Rothana’’, ‘’Khalas’’, ‘’Zaghloul’’, and ‘’Aref’’ cvs. Monoolein (peak 80) was detected in its highest concentration in ‘’Rhothana’’ (10.42 mg/g) and ‘’Zaghloul’’ (9.8 mg/g) and it was previously detected in seeds of Saudi Ajwa cv^45^. Monoolein was. Monoglyceryl fatty acid esters are reported for their antimicrobial and antifungal effects^46^, and whether seeds rich in esters such as ‘’Khalas’’ and ‘’Lolo’’ exhibit potential antimicrobial or food preservative action should be further examined based on these profiling results. Lauric acid glyceryl ester^46^ was identified as peak 92 in ‘’Breem’’ and ‘’Zaghloul’’ cvs. though at trace levels (1.5 mg/g).

#### Sugars

Sugars represented the second most abundant class in most cvs. represented by a total of 18 peaks reaching up to 128.9 mg/g in ‘’Khalas’’ date palm so can be a good choice as a rich feed material with high energy content. In contrast, the least sugar content was detected in ‘’Nabout Seif’’ and ‘’Barhi’’ (0.1 mg/g and 0.16 mg/g, respectively). Monosaccharides were detected in 11 peaks, with glucose, fructose, and mannose, as the most abundant forms, and in accordance with previous results^15,47^. Disaccharides were annotated in 4 peaks, with sucrose (peak 79) as most dominant disaccharide in 6 cvs., including ‘’Zaghloul’’, ‘’Aref’’, ‘’Breem’’, ‘’Rothana’’, ‘’Omeldehn’’, ‘’Khalas’’, with the highest level in ‘’Zaghloul’’ seed (33.39 mg/g). Nevertheless, sucrose was not detected in ‘’Barhi’’, ‘’Nabout Seif’’, and ‘’Farsi’’ cvs. In a previous report on 18 cvs. from various Arab regions, sucrose was absent in all samples, except in ‘’Khalas’’ seeds^12^, and our results indicate that mono sugars amount more for date seed calorie, and likely sensory attributes than di-sugars, and in agreement with results in fruits^48^. The sugar content of date seeds can introduce them as natural sweeteners in food products and syrups in addition to enhancing taste and products acceptability^49^.

#### Sugar alcohols

Sugar alcohols are class of carbohydrates that have hydroxyl group substituting the aldehyde group of monosaccharides^50^ termed as diabetic sugars as they do not tend to increase blood sugar level contrary to sugars^51^. Sugar alcohols (7) were identified in the tested samples where the highest content was detected in ‘’Khalas’’ date palm seed (26.7 mg/g), with myo-inositol representing the major detected sugar alcohol (24.78 mg/g). Like sucrose, myo-inositol was not detected in ‘’Barhi’’, ‘’Nabout Seif’’, and ‘’Farsi’’ cvs., suggestive for their similar sugar and sugar alcohol profiles. Sugar alcohols exhibit potential food value due to their low calorific value and slow absorption from the intestine posing them as natural sweeteners for obese patients^50^. Being the most rich in sugar alcohols, “Khalas” cv. can be suggested as a source of these sugars^49^ .

#### Organic acids

Organic acids were detected at high levels in ‘’Aref’’, ‘’Breem’’, and ‘’Zaghloul’’ cvs. versus (vs.) much lower levels in ‘’Barhi’’, ‘’Nabout seif’’, and ‘’Farsi’’. Organic acids contribute to the flavor of powdered date seeds especially as coffee substitute^6,52^, in addition to their preservation action^4^. A total of 17 organic acids were identified in examined cvs. for the first time with the highest level reported for tiglic acid (peak 2) in ‘’Breem’’, ‘’Aref’’, and ‘’Zaghloul’’ date palm seeds detected at 27.3, 27.1, and 25.4 mg/g, respectively (Table S1). Other identified organic acids included lactic acid (peak 1), 3-hydroxypropionic acid (peak 10), succinic acid (peak 21) and fumaric acid (peak 44). Lactic, succinic, methyl succinic, fumaric, 3-hydroxypropionic, 4-hydroxybutyric and methyl maleic acids were previously reported in ‘’Majdool’’ cv^15^. Short-chain acids, e.g., lactic and fumaric acids are significant components in roasted coffee and participate in the sourness of coffee blends^53^. Variation in the content of organic acids among cvs. can be attributed to physiological, environmental and genetic factors which influence the enzymes catalyzing their synthesis and degradation in date seeds^54^.

#### Amino acids

A high content of amino acids was previously reported in date palm fruits^55^. In the current study, pyroglutamic acid (peak 33) and L-threonine (peak 34) were detected for the first time in date palm seed cvs. L-threonine is an essential amino acid with several roles, *i.e.*, growth and cell proliferation, immunostimulant, prebiotic, modulating impaired lipid metabolism in addition to gut homeostasis^56,57^ detected though at low levels 0.01 to 0.8 mg/g, with highest levels in ‘’Aref’’, ‘’Breem’’, and ‘’Rothana’’ at 0.6–0.8 mg/g, respectively. L-threonine content in ‘’Aref’’ was considerably low compared to its level reported for Soy bean (2.4 mg/g)^58^. Pyroglutamic acid (peak 33), is a biomolecule identified as a terminal point in protein chains, collagen, enzymes, and antibodies^59^. It was detected at the highest level in ‘’Breem’’ (0.35 mg/g), ‘’Rothana’’ (0.33 mg/g), ‘’Aref’’ (0.28 mg/g), and ‘’Zaghloul’’ (0.28 mg/g) date palm seeds. Moreover, it is used to exhibit natural taste modifier effects to impart sour and umami flavors^60^. These findings are consistent with previous reports for its presence in ‘’Majdool’’ cv^15^.

#### Steroid/terpenoids

Steroids and terpenoids are chemical classes previously reported in date pits^6,15^. *β*-Sitosterol (peak 98) was identified at different levels ranging from 0.03–4.0 mg/g with the highest content in ‘’Rothana ‘’ and ‘’ Breem ‘’ at 3–4 mg/g. These levels are much higher compared to concentration of *β*-Sitosterol in some natural sources e.g., wheat (0.29–0.49 mg/g), rye (0.36–0.61 mg/g), rice (0.38 mg/g) and pea (0.41 mg/g)^61^. *β*-Sitosterol was previously reported from the leaves of Saudi date palm^62^, but is first time to be reported in date palm seeds. Other terpenoids exemplified by *β*-caryophyllene, *β*-elemene, and ursolic acid were detected for the first time in date palm seeds adding to their food properties. Nor-epiandrosterone was found at high level in ‘’Aref’’ seed (15.9 mg/g), of potential as anabolic steroid to increase the muscle size, weight and general performance^63^ as well as antitumor effect^64^. Androgenic steroids were reported in some plants e.g. wheat, *Nicotiana tabacum* and *Inula helenium* ranging from 2.29 × 10^− 6^ mg/g to 5.73 × 10^− 6^ mg/g)^65^ showing lower levels than that detected in ‘’Aref’’ seeds.

#### Phenolic acids

Phenolic acids are reported in both date palm seeds^11^ and fruits^6,7,66^. In the current study, ‘’Omeldehn’’ cv. possessed the highest phenolic acid content followed by ‘’Zamli’’ at 0.32% and 0.21%, respectively. In addition, 4-methoxymandelic (peak 38), protocatechuic (peak 45) and sinapic acids (peak 49) were identified in all cvs. Protocatechuic (peak 45) was reported for its antioxidant, antimicrobial and anti-inflammatory actions so widely used as safe additive to animal feed that can reduce extensive use of antibiotics in breeding^67^. Protocatechuic acid was previously reported in date palm seed^6,11^, while sinapic acid was previously identified in Saudi and Egyptian date fruits^7,66^, and first time to be reported in seeds.

#### Flavonoids

Compared to flavonoids richness in date fruit^66^, they were detected at minor levels, though considering that GC-MS is not optimized for profiling of polar phenolics such as flavonoids. Date seeds are considered a good source of flavonoids, especially catechins^6,11^. In accordance with these reports, catechin (peak 86) was identified in all cvs. and at highest levels in ‘’Zaghloul’’ cv. (1.6%) followed by ‘’Hasawi’’ cv. (1.48%). Catechin was reported for a wide spectrum of bioactivities such as a potential antioxidant^68^. Profiling of flavonoids using LC-MS should now follow to determine whether ‘’Zaghloul’’ cv. present potential source of catechins and aid to identify other forms.

#### Miscellaneous

In addition to the previous phytochemicals, alcohols, aldehyde, and hydrocarbons were detected in date palm seeds. Five alcohols were detected, with glycerol (peak 19) as major component detected at high levels in ‘’Breem’’, ‘’Aref’’, ‘’Zaghloul’’, and ‘’Rothana’’ at 27.8, 17.0, 11.7, and 10.7 mg/g, respectively. Decanal (peak 17) was the only aldehyde found mainly in ‘’Rothana’’, ‘’Breem’’, ‘’Zaghloul’’, and ‘’Aref’’ seeds though at trace levels, Table S1. Likewise, hydrocarbons were detected at trace levels and represented by octacosane (peak 85) and hentriacontane (peak 90). Octacosane (peak 85) showed antidiabetic, antioxidant and anti-inflammatory activities in previous reports^69^ and was detected before in date palm leaves^62^, while hentriacontane (peak 90) was reported for its antitumor properties^69^.

### Multivariate data analyses of primary metabolites in date seeds cvs

**Fig. 3:**
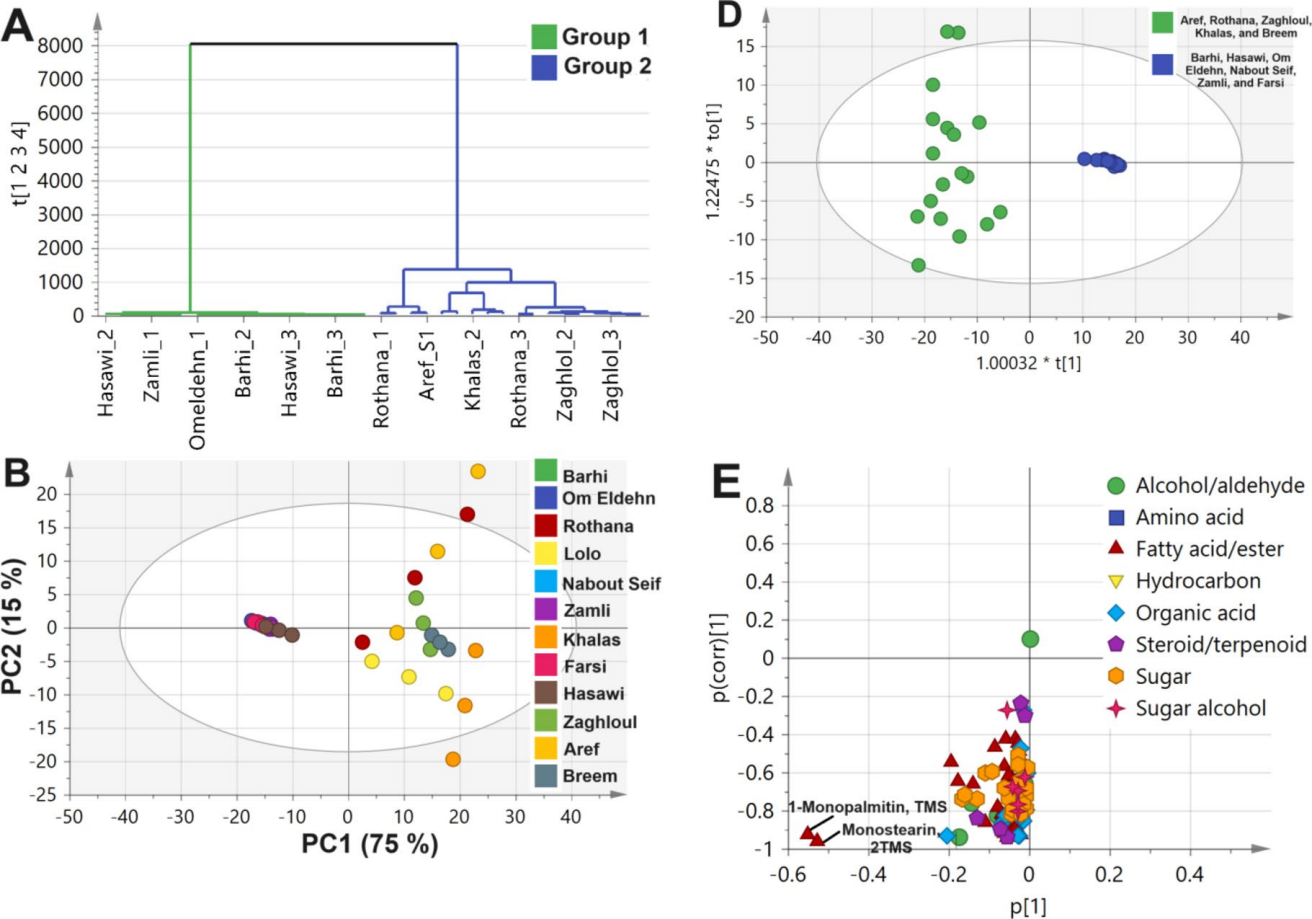

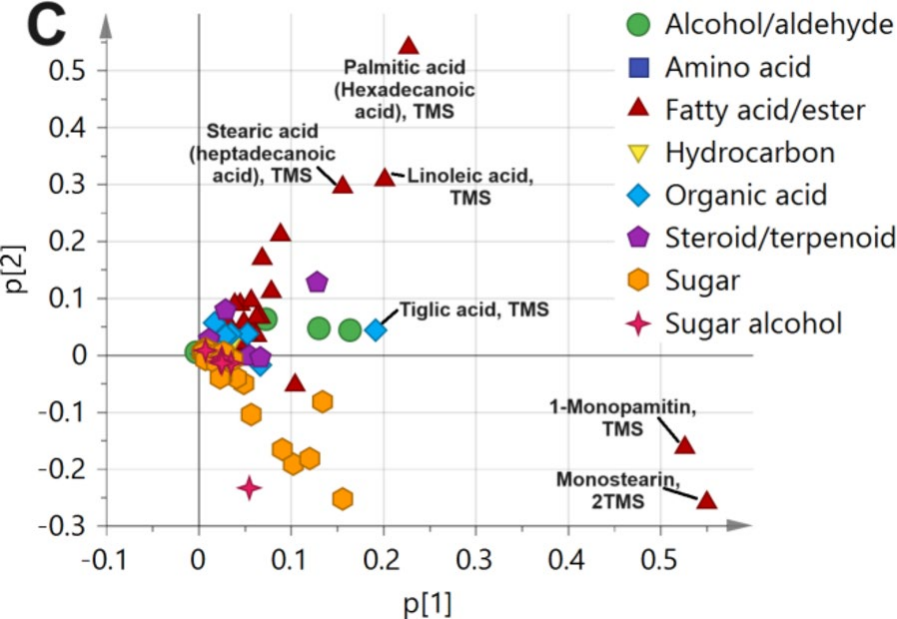
Date palm seed cvs. dataset modelling of primary metabolites as analyzed by gas chromatography combined with mass spectrometry (GC-MS) post-silylation. Unsupervised data modelling of the 12 cvs. (**A**) Hierarchical cluster analysis (HCA), (**B**) Principal component analysis (PCA) score plot, and (**C**) PCA loading plot. Supervised orthogonal partial least squares discriminant analysis (OPLS-DA) modelling of Group 1 against Group 2 varieties as appeared in HCA model (**D**) Score plot, (**E**) OPLSA-DA S-plot. Group 1 consisted of ‘’Bahri’’, ‘’Hasawi’’, ‘’Omeldehn’’, ‘’Nabout Seif’’, ‘’Zamli’’, and ‘’Farsi’’ varieties, while Group 2 included ‘’Aref’’, ‘’Breem’’, Khalas, ‘’Lolo’’, and ‘’Zaghloul’’.

Following metabolites profiling of primary metabolites, *i.e.*, sugars, fatty acids, amino acids, etc., by GC-MS post-silylation, it was of value to model GC-MS dataset considering its complexity of 12 date palm seed cvs., alongside a variable of 101 detected metabolites. The models included both unsupervised (HCA and PCA) and supervised (OPLS-DA), Fig. 3.

#### Unsupervised HCA and PCA multivariate data analyses of primary metabolites GC-MS dataset

GC-MS dataset was modelled initially using HCA for sample classification as shown in Fig. 3A. HCA dendrogram showed 2 main groups, where Group 1 encompassed ‘’Barhi’’, ‘’Hasawi’’, ‘’Omeldehn’’, ‘’Nabout Seif’’, ‘’Zamli’’, and ‘’Farsi’’ cvs., while Group 2 included other cvs. Similar segregation pattern was observed in the case of PCA model in which cvs. of Group 1 were clustered on the left side away from Group 2, with specimens positioned on the opposite side. PCA model explained 75% of the total variance, Fig. 3B. PCA Loading plot revealed for metabolites mediating for such clustering pattern. The cluster of Group 2 was associated with fatty acids/esters (e.g., palmitic acid, linoleic acid, stearic acid, 1-monopalmitin, and monostearin), in addition to organic acids (e.g., tiglic acid), and suggestive that ‘’Aref’’, ‘’Breem’’, ‘’Khalas’’, ‘’Lolo’’, and ‘’Zaghlol’’ cvs. were richer in nutrients. In contrast, remaining cvs. encompassed in Group 1 did not show any specific markers, Fig. 3C.

#### Supervised OPLS multivariate data analysis of primary metabolite GC-MS dataset

Based on samples’ classification results observed in HCA (Fig. 3A) and PCA (Fig. 3B), both Groups were further modelled using OPLS-DA which aids in identifying markers more accurately^70^. The OPLS-DA score plot, Fig. 3D, revealed that both Groups were well separated along the x-axis negative and positive side. Furthermore, OPLS-DA S-plot revealed fatty acyl esters, *i.e*., 1-monopalmitin, and monostearin, were more enriched in cvs. within Group 2, Fig. 3E, and in agreement with quantification results Table S1, and PCA results. Mono- and diacylglycerol of fatty acids are used as food emulsifiers belonging to the type E471 modifying the techno-functional qualities of different foodstuff^71^ posing ‘’Aref’’, ‘’Breem’’, Khalas, ‘’Lolo’’, and ‘’Zaghlol’ as potential sources of multifunctional food components for valorization purposes of date seeds in these cvs. Furthermore, Figure S1 showed the optimization and validation parameters for OPLS-DA modelling of post silylation GC-MS dataset of Group 1 against Group 2 varieties as appeared in HCA model. The diagnostic metrics R^2^Y and Q^2^ as function of number of principal components is shown in Figure S1A, while permutation test (*n* = 20) showed negative Q^2^ intercept value in Figure S2A, and CVANOVA is demonstrated to assess model statistical significance (*p* < 0.05). R^2^, Q^2^, and permutation test showed acceptable variance coverage and prediction power above 0.9 and *p* value significant less than 0.05.

### Aroma profiling in date palm Cvs. *via* headspace solid phase microextraction (SPME) coupled to GC-MS

**Fig. 4:**
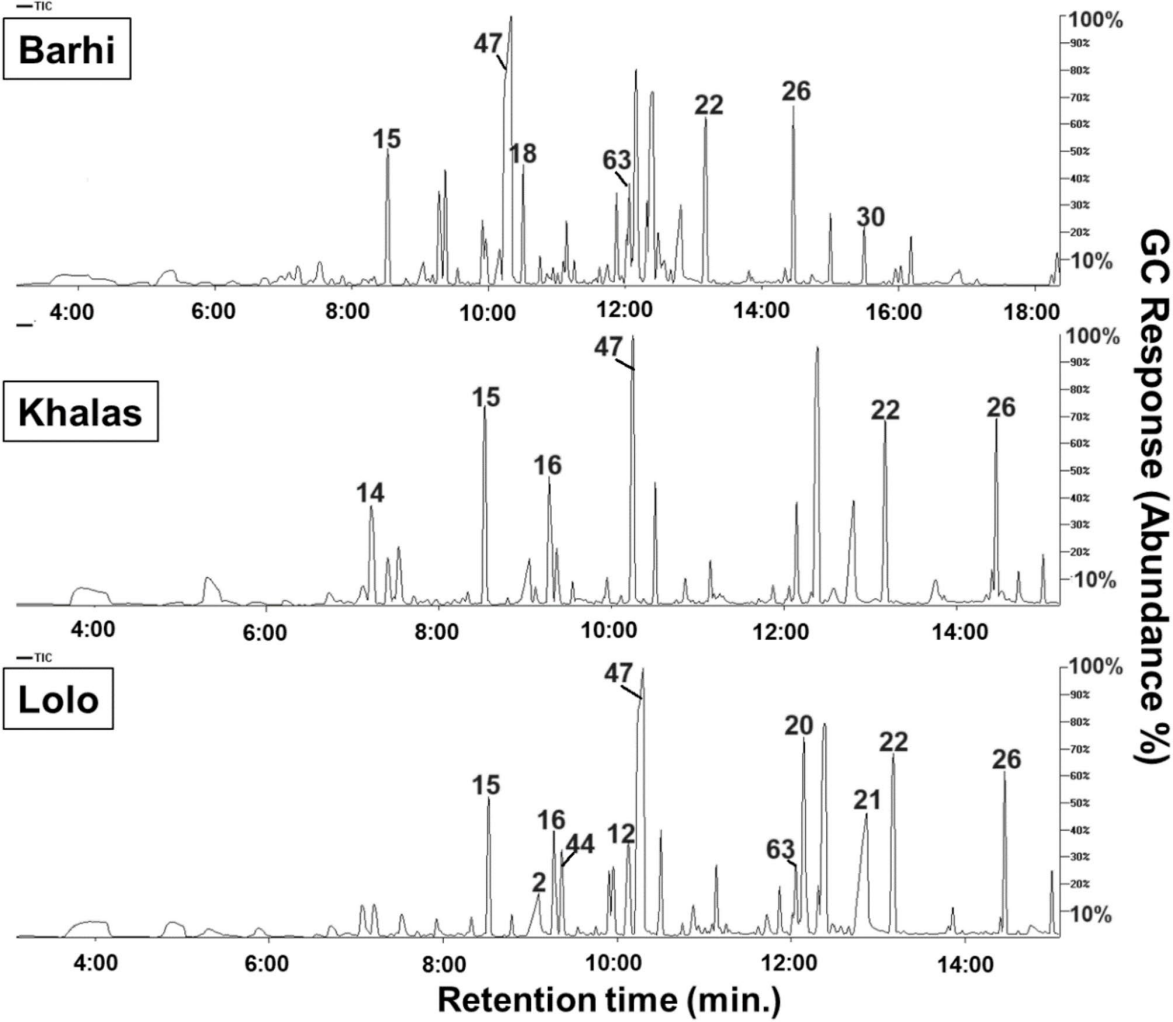
Aroma total ion chromatograms (TIC) of some representative cultivars of roasted date palm (*P. dactylifera* L.) seed cvs. ‘’Barhi’’, ‘’Khalas’’, and ‘’Lolo’’ as analyzed using head space-solid phase micro-extraction coupled with gas chromatography and mass spectrometry (HS-SPME/GC-MS). The peak numbers denote for the identified metabolites listed in Table S2.

Compared to extensive reports on date fruits aroma, few studies have been reported on its seed aroma profile especially being used a coffee substitute^15^. Out of the 12 investigated date palm seeds cvs., 8 cvs., *i.e.*, ‘’Barhi’’, ‘’Omeldehn’’, ‘’Rothana’’, ‘’Lolo’’, ‘’Nabout’’ ‘’Seif’’, Khalas’’, ‘’Farsi’’, and ‘’Breem’’, were selected for roasting process to intensify their aroma and further analysed using SPME/GC-MS. In contrast to Tunisian cvs. which showed previously only 45 VOCs^72^, a total of 65 metabolites were detected in the current study as listed in Table S3. Volatiles belonged to acids (3), alcohols (7), aldehyde (2), esters (6), fatty acids/esters (19), monoterpene hydrocarbon (4), phenol/ether (9), sesquiterpene hydrocarbon (13), in addition to furan (1), and ketone (1). Interestingly, the most abundant classes were esters, fatty acids/esters, Table S3 & Fig. 4, and in agreement with nutrient results, Table S1.

**Fig. 5:**
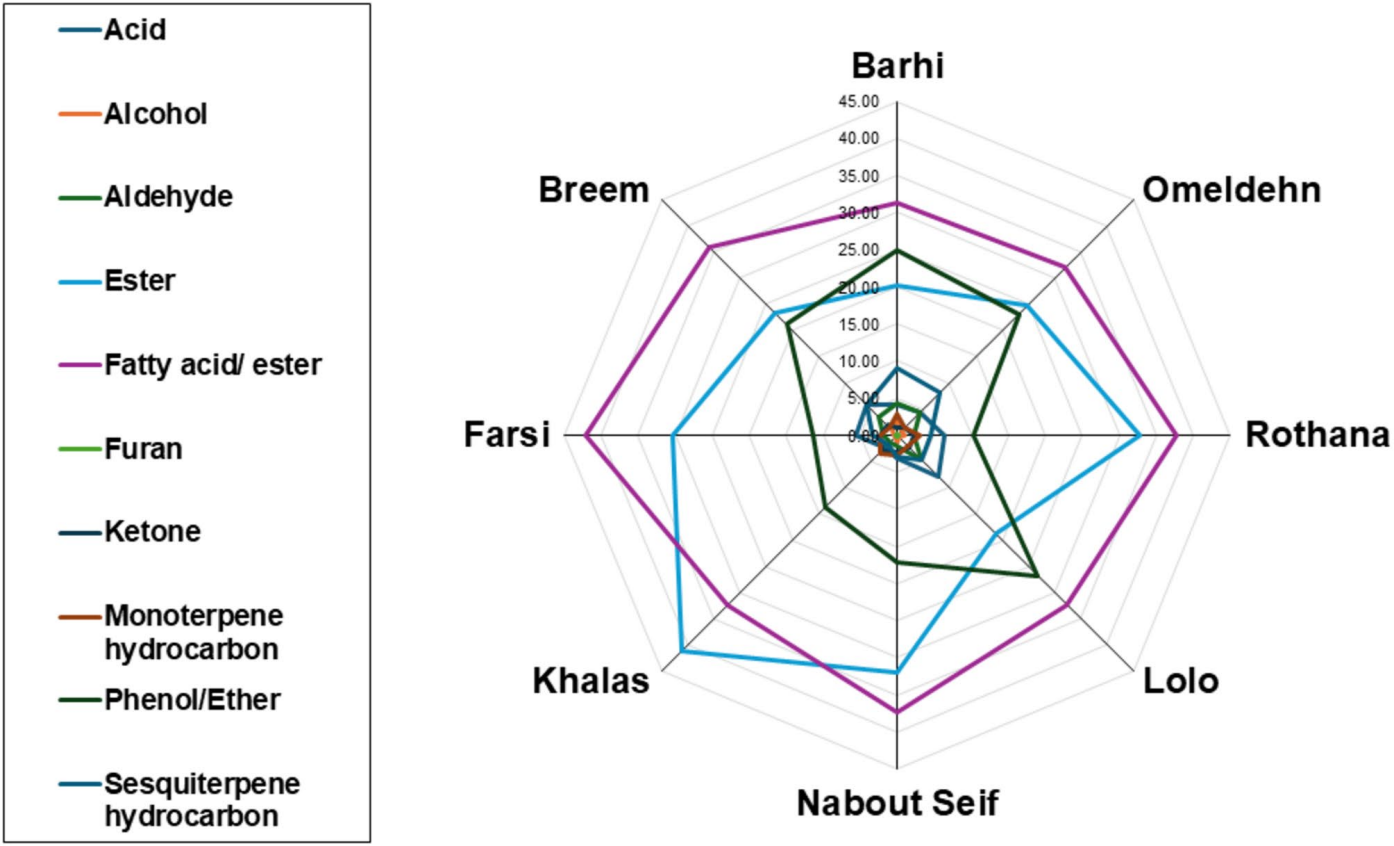
Relative abundances (%) of the various volatile aroma classes detected in roasted date palm (*P. dactylifera* L.) seed cvs. as analysed using SPME coupled with GC/MS. The relative abundances of each class and identified metabolites are listed in Table S2.

The total abundance of all aroma classes is depicted in Fig. 5 revealing that esters and fatty acids/esters followed by phenols/ethers dominated date palm seed aroma. Fatty acids/esters were the most abundant in all samples, except for ‘’Lolo’’ and ‘’Barhi’’, at which phenols/ethers were more abundant. The following subsections shall discuss the various classes in detail.

#### Fatty acids/esters

Fatty acids/esters amounted for the most abundant class represented by 19 metabolites detected at 31.3–42.1% of the total VOCs abundance, Table S3. Major forms included dodecanoic acid (lauric acid), methyl ester (peak 20), dodecanoic acid, ethyl ester (peak 22), and n-hexadecanoic acid (palmitic acid) (peak 21).

It should be noted that most of the identified fatty acids were of the saturated type represented by 15 out of the 19 (79%). These results were in agreement with previous reports that most of the seed oil is rich in saturated fatty acids (e.g., lauric, myristic, and palmitic acids)^10^, and likely to be observed in its aroma profile.

#### Esters

High levels of esters were detected in date palm seed aroma represented by 6 volatiles, Table S3. The highest ester level was detected in ‘’Khalas’’ cv. vs. lowest in ‘’Lolo’’ cv. *cis*-3-Hexenyl acetate (peak 14) was the most abundant ester reaching 21.1% in ‘’Khalas’’ and likely mediate a pleasant fruity aroma of this cv^73^. Besides, octanoic acid was among the most abundant metabolites detected as either methyl (peak 15) or ethyl (peak 16) esters ranging from 7.1–12.4%, and 2.4–5.8%, respectively. Also, decanoic acid, methyl ester (peak 18) was a major ester at 5.1% in ‘’Rothana’’ cv. Collectively, it is evident that fatty acids alongside their oxidative products likely generated upon roasting that accounted for date seed aroma^74^.

#### Phenols/ethers

Phenols/ethers represented the third most abundant class. The highest level was in ‘’Lolo’’ vs. lowest level was in ‘’Rothana’’ cv, Table S3. Nine volatiles were detected, of which anethole (peak 47) was the most abundant accounting for 9.1–23.3% (*ca.* 2.5-fold between the lowest and highest level) of seeds’ aroma, and likely to impart a characteristic herbaceous aroma^75^. In comparison, estragole (peak 44) and myristicin (peak 51) were detected at 0.5–3.5% (*ca.* 7.0-fold) and 0.02–1.5% (*ca.* 75-fold), respectively. Anethole is a key flavor in several spices and herbs including^76^, and is reported in date seed for the first time. Whether anethole contributes to date seed aroma perception has yet to be examined.

#### Sesquiterpene hydrocarbons

Sesquiterpene hydrocarbons (peaks 53–65) were represented by a total of 13 peaks, Table S3, ranging from 1.8–9.0%, with highest levels detected in ‘’Barhi’’ cv. at 9.0%. Such high levels were attributed to *α*-curcumene (peak 62) and *α*-guaiene (peak 63) accounting for 1.9 and 3.1%, respectively.

#### Acids & aldehydes

Total acids amounted for minor amounts in date seed aroma of 2.5–7.8 represented by 3 acids. The highest level was detected in ‘’Lolo’’ vs. lowest in ‘’Khalas’’ cv, Table S3. Octanoic acid (peak 2) was the major form at 4.5%. Such acids are most likely formed *via* oxidation of saturated long fatty acids^77^ as previously discussed.

Aldehydes were likewise detected at minor levels (1.4–4.7%) represented by *p*-anisaldehyde (peak 11) and (*E*)-cinnamaldehyde (peak 12). *p*-Anisaldehyde was found highest in ‘’Omeldehn’’ and ‘’Barhi’’ cvs. at 3.5% and 2.8%, respectively, whereas (*E*)-cinnamaldehyde was detected in ‘’Lolo’’ at 2.3%. Both volatiles belong to phenylpropanoids characterized by their unique aroma and to improve scent in these date seed cvs. though detected at low levels but have strong odour threshold^78^. The absence of short-chain aldehydes in aroma composition (e.g., hexanal, propanal, …etc.) suggested that no rancidity occurred to these seeds being enriched in fatty acids^79^.

#### Miscellaneous

Volatile classes detected at trace levels included alcohols (0.4–1.9%), ketones (1.1–3.1%), furans (0.1–0.2%), and monoterpene hydrocarbons (1.4–3.4%). *α*-Methylstyrol (peak 4, 0.9%) and D-limonene (peak 41, 3.0%) were the most abundant in alcohols and monoterpene hydrocarbons, respectively.

### Multivariate data analyses of aroma profile in roasted date seed Cvs. following SPME/GC-MS

Considering differences in aroma composition observed from chromatograms inspection, Fig. 4, modelling of aroma profile in date cvs., *i.e.*, ‘’Aref’’, ‘’Barhi’’, ‘’Breem’’, ‘’Khalas’’, ‘’Lolo’’, ‘’Nabout Seif’’, ‘’Omeldehn’’, and ‘’Rothana’’ was attempted in both unsupervised (e.g., HCA and PCA) and supervised (e.g., OPLSA-DA), and to compare with classification results of nutrients, Fig. 3. In addition, OPLS-DA model validation was performed by permutation test showing goodness of fit represented by R^2^ and prediction power (Q^2^)^80^.

#### Unsupervised HCA and PCA multivariate data analysis of date seed aroma dataset

**Fig. 6:**
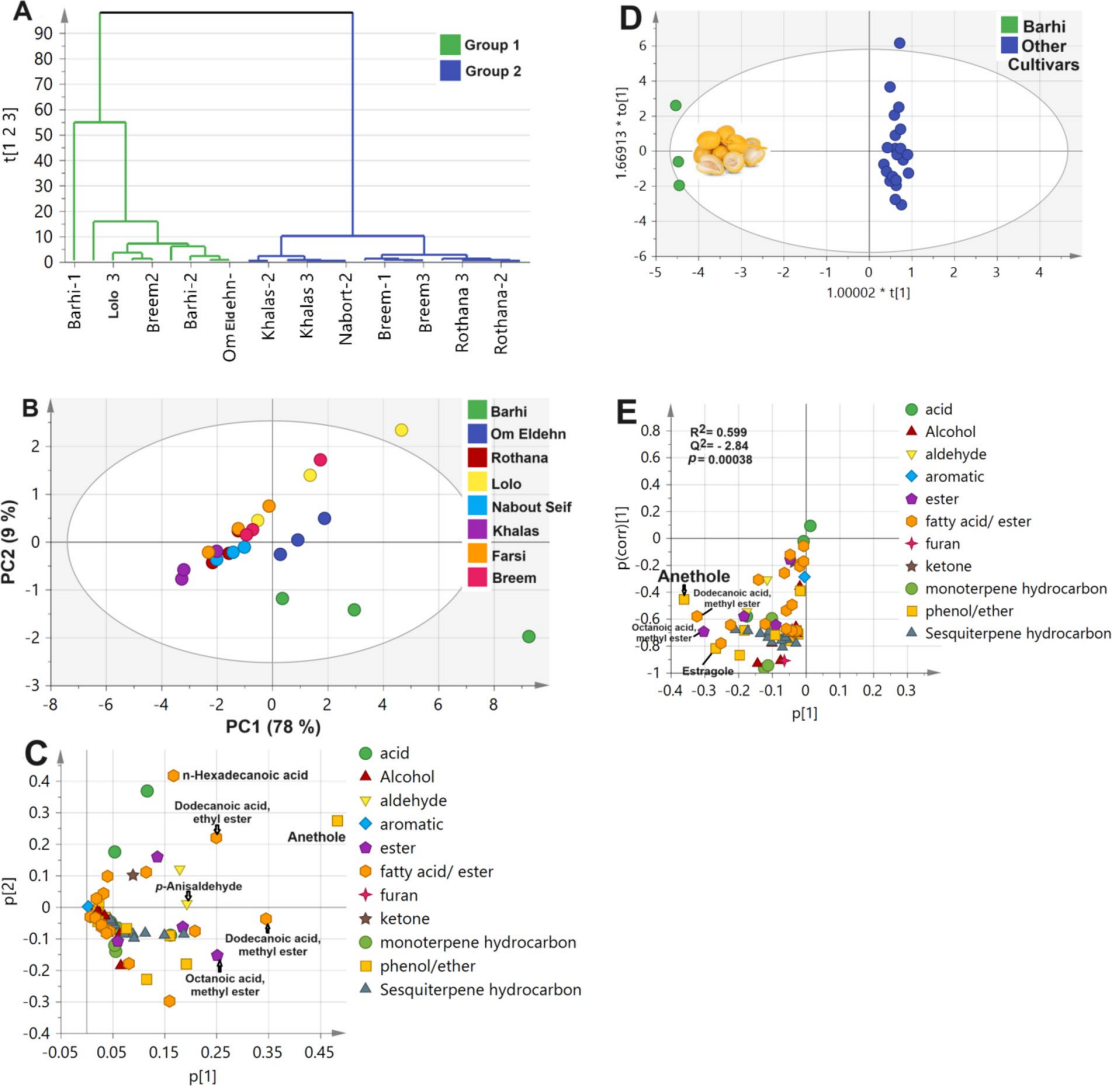
Date palm seed cvs. dataset modelling of volatile constituents as analyzed by head space-solid phase micro-extraction coupled with gas chromatography and mass spectrometry (HS-SPME/GC-MS). Unsupervised data modelling of the 8 cvs, including ‘’Barhi’’, ‘’Omeldehn’’, ‘’Rothana’’, ‘’Lolo’’, ‘’Nabout Seif, Khalas’’, ‘’Farsi’’, and ‘’Breem’’. (**A**) Hierarchical cluster analysis (HCA), (**B**) Principal component analysis (PCA) score plot, and (**C**) PCA loading plot. Supervised orthogonal partial least squares discriminant analysis (OPLS-DA) modelling of ‘’Barhi’’ cultivar against other samples (**D**) Score plot, (**E**) OPLSA-DA S-loading plot showing model validation indices by permutation test at *p* < 0.05.

HCA dendrogram of aroma seeds’ dataset revealed for 2 main clusters, in which ‘’Barhi’’, ‘’Omeldehn’’, and ‘’Lolo’’ cvs. appeared as unique group from other samples, Fig. 6A, which is in partial agreement with primary metabolites modelling shown in Fig. 3A. Also, clustering of ‘’Breem’’, ‘’Khalas’’, and ‘’Aref’’ in the cluster group was comparable to primary metabolites modelling results. Both groups segregated along PC1 accounting for 78% of the total variance (87%) as appeared in PCA score plot, Fig. 6B. It is worth mentioning that model covered sample variances by more than 85%, primarily along PC1, accounting for 75 and 78%, respectively. Furthermore, PCA loading plot revealed aroma peaks mediating for segregation to include anethole (peak 47), n-hexadecanoic acid (peak 21), methyl and ethyl esters of dodecanoic acid (peaks 20/22), and octanoic acid, methyl ester (peak 15), Fig. 6C. These aromas were found to be more enriched in the first group, including ‘’Barhi’’, ‘’Omeldehn’’, and ‘’Lolo’’ cvs. and to likely impart improved aroma of these cvs. to be more targeted for food applications compared to other examined cvs. which has yet to be confirmed using sensory analysis. Barhi is a premium cv. and its enrichment in anethole, and distant segregation in the model can account for its favored sensory properties.

#### Supervised OPLS-DA multivariate data analysis of aroma profile dataset

Following the unsupervised modelling revealing segregation of ‘’Barhi’’ cv. as the most distant cv. in the lower quadrant of score plot and as outlier, Fig. 6B, supervised modelling was applied by modelling ‘’Barhi’’ cv. in one class against other cvs. especially ‘’Barhi’’ is a premium cv. in dates and be of interest to identify aroma marker distinguishing this cv. The OPLS-DA score plot showed a distant segregation of ‘’Barhi’’ on the negative side away from other cvs. on the positive counterpart, Fig. 6D, as expected. Various aroma metabolites appeared in the corresponding S-loading plot (Fig. 6E) to be associated potentially with ‘’Barhi’’ cv., including anethole (peak 47), estragole (peak 44), dodecanoic acid, methyl ester (peak 20), in addition to octanoic acid, methyl ester (peak 15), and indicating its rich aroma composition especially in anethole as key flavor in date seed. The model validation by permutation test (*n* = 20) implied a significant model (*p* < 0.05) and no data overfit supported with R^2^ = 0.6 and Q^2^ = − 2.8 indicating the model’s goodness of fit and high prediction power, respectively^80^, Fig. 6E.

Moreover, Figure S2 demonstrated optimization and validation parameters for supervised OPLS-DA modelling of ‘’Barhi’’ VOCs profile against Group 2 including all other varieties. Figure S2A showed the diagnostic metrics R^2^Y and Q^2^ as function of number of principal components. In addition, the permutation test (*n* = 100) showed negative Q^2^ intercept value (Figure S2B), while Figure S2C demonstrated CVANOVA and confirmed the model’s statistical significance (*p* < 0.05).

### Minerals profiling in seeds of different date palm Cvs

**Fig. 7:**
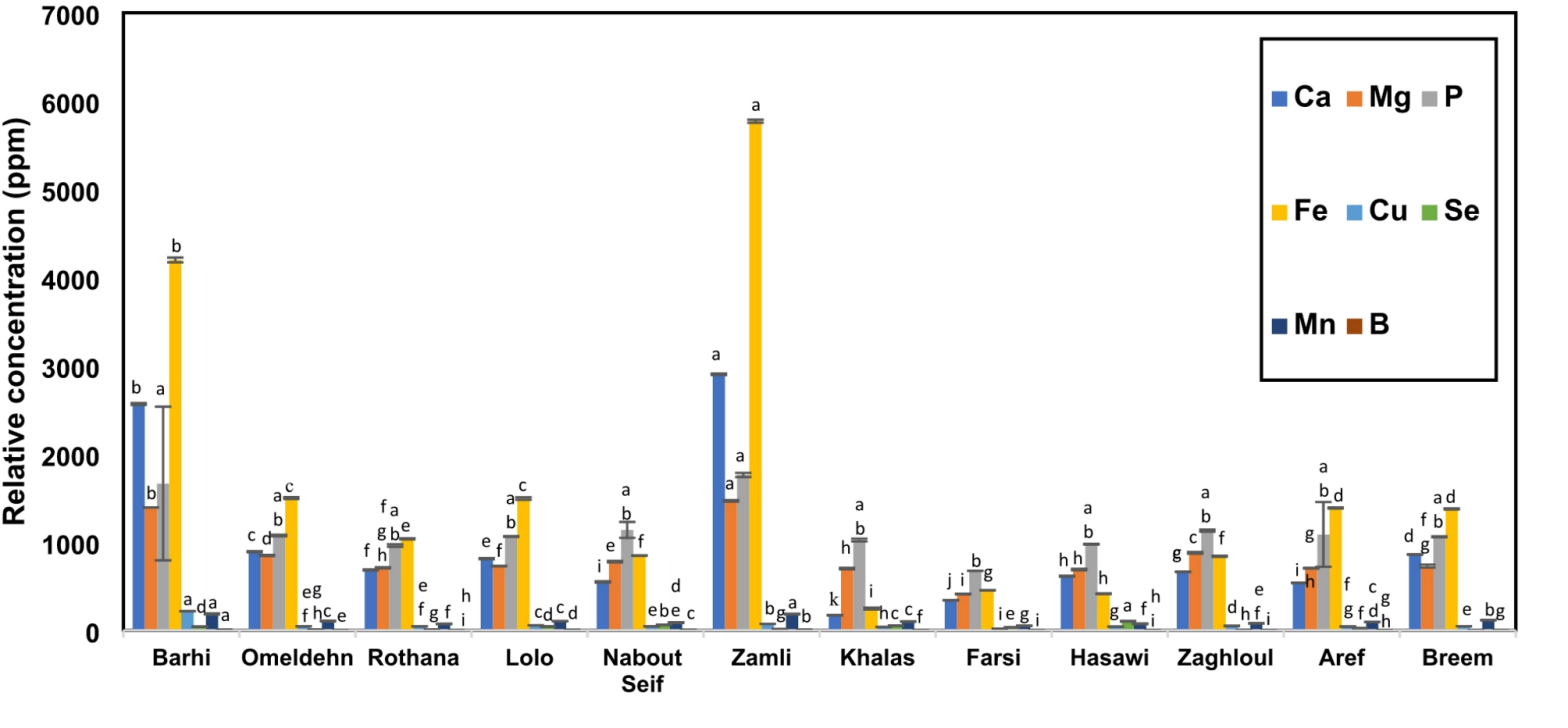
Mineral content, including macro-elements; iron (Fe), selenium (Se), copper (Cu), manganese (Mn) and boron (B) and macro-elements; calcium (Ca), magnesium (Mg) and phosphorus (P) of *P. dactylifera* L. seeds of different cvs. represented as ppm ± SD. Statistical analysis is carried out by one-way ANOVA, where cvs. that do not share the same letter in each element are significantly different at *p* < 0.05.

Minerals targeted in this assay included macro-minerals, *i.e.*, Ca, Mg and P and micro-minerals, *i.e.*, Fe, Se, Cu, Mn and B, yet to be examined as a part of this study in date seed cvs. The concentrations of the eight tested minerals in date palm cvs. are represented in Table S4 and Fig. 7.

Ca was detected at the highest level in ‘’Zamli’’ (2902.3 ppm), much higher than that recorded in the Bahraini palm seeds that ranged from (64.51 ppm in ‘’Khaseeb’’ cv. up to 113.3 ppm in the Bahraini ‘’Khalas’’)^34^. Ca is one of the most abundant elements in the human body which is mainly precipitated in bones and teeth^26^. It also plays a critical role in cell membrane function and intracellular transmissions^26^. Mg was detected likewise at highest levels in ‘’Zamli’’ and ‘’Barhi’’ (1469.0 and 1393.0 ppm, respectively) while detected in almost half this amount ranging from 705.3 to 779.3 ppm in ‘’Aref’’, ‘’Rhotana’’, ‘’Breem’’, ‘’Khalas’’, ‘’Lolo’’, and ‘’Nabout Seif’’. Like Ca, Mg showed higher levels in the Egyptian seeds compared to the Bahraini originated cvs. (613.1 to 695.5 ppm)^34^ and showed similar levels to Saudi cvs. (780.22 ppm)^29^. Mg is important for the function of many enzymes as well as participation in muscle contraction process, DNA and RNA replication and intercellular signals^27,33^.

P showed its highest levels in ‘’Zamli’’ and ‘’Barhi’’ cvs. Enrichment in date seed in Ca and P in date seeds warrant for further bioassays for their effects on bone mineralization, e.g., in vitro alkaline phosphatase activity assay and measuring in vivo bone forming potential^81^. Fe was abundant in ‘’Zamli’’ and ‘’Barhi’’ at 5771 and 4199 ppm, respectively. The highest concentration detected in all micro-elements was for iron and in accordance with a previous study on seeds of Emirati cvs^28^. Iron is one of the most essential elements for the vitality of the human body contributing to the development of the immune system, erythropoiesis, and formation of cellular energy^26^.

Selenium (Se) exhibited a wide range of concentrations among different cvs. found at highest level in ‘’Hasawi’’ (104.05 ppm). Se is vital for some enzymes inside the body that act as antioxidant, antiviral, and immunomodulatory functions^26^. Cu was determined at highest level (218.7 ppm) in ‘’Barhi’’ vs. least in Farsi (20.49 ppm). Cu plays a supportive role in neuronal health, proper brain function, oxidative defense and mood balance^26^. Mn showed similar pattern being found at highest level in ‘’Barhi’’ at 186.0 ppm.

Comparison among examined seeds, ‘’Zamli’’ and ‘’Barhi’’ exclusively exhibited the highest content of Ca, Mg, P, Mn, Fe, Cu, and B, while ‘’Hasawi’’ possessed the highest amount of Se. Estimation of mineral content showed differences in their concentration compared to previous studies^28,34^. These variations may be attributed to different harvesting time, type of soil, mineral content of soil, irrigation water, and other environmental aspects as well as genetic differences^28^.

## Conclusion

The current study explored for the first time the metabolites and minerals heterogeneity in major cvs. of date palm seeds collected from Egypt as a main producer of date fruit worldwide. Non-volatile and volatile metabolomes were targeted to uncover new applications of date palm seeds and extend our previous investigations on date fruit. Date palm seeds showed richness in key nutrients such as essential fatty acids (e.g., omega-9 and − 6 fatty acids) and amino acids (e.g., pyroglutamic acid and L-threonine) adding to their feed value and valorization purposes. Particularly, ‘’Khalas’’ cv. showed the highest abundance of monoacylglycerols (e.g., 1-monopalmitin and monostearin) suggesting its incorporation in food and pharmaceutical industries as a natural emulsifier to improve texture, consistence and final appeal of wide range of products. ‘’Khalas’’ cv. also can be a safe sweetener candidate based on its high sugar alcohols content. In addition, volatile aldehydes (e.g., cinnamaldehyde and *p*-anisaldehyde) and ethers (e.g., anethole) were first time to be reported in date seed, found mostly enriched in ‘’Omeldehn’’, ‘’Barhi’’, and ‘’Lolo’’ cvs. posing them as potential cvs. to be used as a coffee substitute considering their improved aroma composition. Moreover, ‘’Barhi’’ cv. appeared as a distinctive and premium cv. in the context of aroma composition as confirmed by unsupervised and supervised modelling attributed to the richness in the fragrant phenylpropanoids anethole and estragole, in addition to dodecanoic acid methyl ester, and octanoic acid, methyl ester indicating its rich aroma composition with key flavor components. Furthermore, assay of minerals in date palm seeds for cvs. grown in Egypt showed higher levels of minerals, *i.e.*, Fe, Ca, and P, Mn compared to seeds of Emirati and Bahraini cvs. ‘’Barhi’’ and ‘’Zamli’’ seeds should be considered as food supplements for their high mineral content and yet to be tested using bioassays. Consequently, ‘’Barhi’’ cv. was exclusively rich in aroma components and possesses high mineral composition. Variation in the phytochemical profile of date cvs. seeds can be attributed to different factors such as their origin, genetic factors, environmental and soil conditions^82^. Secondary metabolite differences should be assessed to affect date seed health benefits and for that LC-MS is more suited for profiling date seed bioactives. Future work including in vitro and in vivo studies should follow for various extracts of these cvs. to investigate bioactivities in comparison to metabolite fingerprint, *i.e.*, antioxidant activity, ... etc. Exploration of richness of by-products like date seeds help in their repurposing in development in functional food and dietary supplements by providing natural and sustainable alternatives.

## Electronic supplementary material

Below is the link to the electronic supplementary material.

Supplementary Material 1

## Data Availability

The datasets used and/or analysed during the current study can be made available from the corresponding author on reasonable request.
