# Supplementary material for: GC-MS based nutritional and aroma profiling of date palm seeds collected from different Egyptian cultivars for valorization purposes
Source: Sci Rep. 2025 May 13;15:16531. doi: 10.1038/s41598-025-00171-7 (PMC12075629; doi:10.1038/s41598-025-00171-7)
Supplement: Supplementary file 1 — Supplementary Material 1 [file 41598_2025_171_MOESM1_ESM.docx]

**Supplementary materials**

# **Supplementary Tables**

**Table S1**: Silylated metabolites in date palm (*P. dactylifera* L.) seed cvs. represented as mean (mg/g) ± standard deviation as analyzed using GC–MS (n = 3)

| Peak no. | RT (min) | RI | Metabolite name |  | Content (mg/g ± SD) | | | | | | | | | | | |
| --- | --- | --- | --- | --- | --- | --- | --- | --- | --- | --- | --- | --- | --- | --- | --- | --- |
|  |  |  |  |  | **Barhi** | **Omeldehn** | **Rothana** | **Lolo** | **Nabout Seif** | **Zamli** | **Khalas** | **Farsi** | **Hasawi** | **Zaghloul** | **Aref** | **Breem** |
| Alcohol/aldehyde | | | |  |  |  |  |  |  |  |  |  |  |  |  |  |
|  |  |  |  |  |  |  |  |  |  |  |  |  |  |  |  |  |
| 3 | 6.51 | 1026 | 1,2-Propanediol, 2TMS |  | 0.02 ± 0 | 0.09 ± 0.06 | 0.11 ± 0.02 | 0.08 ± 0.01 | 0.04 ± 0.01 | 0.14 ± 0.04 | 0.03 ± 0 | 0.04 ± 0.01 | 0.2 ± 0.15 | 0.07 ± 0.01 | 0.08 ± 0.01 | 0.07 ± 0.01 |
| 4 | 6.85 | 1057 | 1,3 Propanediol, 2TMS |  | 0.02 ± 0.01 | 0.14 ± 0.06 | 12.01 ± 1.21 | 9.14 ± 1.48 | 0.03 ± 0.01 | 0.23 ± 0.07 | 12.01 ± 0.85 | 0.03 ± 0.01 | 0.38 ± 0.3 | 18.94 ± 0.41 | 19.1 ± 0.07 | 19.13 ± 0.11 |
| 11 | 8.64 | 1173 | 1,4-Butanediol, 2TMS* |  | 0 ± 0 | 0.01 ± 0.01 | 2.9 ± 0.47 | 1.32 ± 0.08 | 0 ± 0 | 0.01 ± 0 | 1.15 ± 0.15 | 0.01 ± 0.01 | 0.01 ± 0 | 5.13 ± 0.13 | 5.29 ± 0.21 | 4.91 ± 0.05 |
| 19 | 10.45 | 1287 | Glycerol, 3TMS* |  | 0.27 ± 0.18 | 0.8 ± 0.35 | 10.72 ± 2 | 7.99 ± 5.35 | 0.22 ± 0.07 | 0.46 ± 0.1 | 4.74 ± 1.19 | 1.88 ± 2.02 | 0.62 ± 0.22 | 11.74 ± 4.26 | 16.99 ± 0.19 | 27.84 ± 4.69 |
| 91 | 28.61 | 3017 | 1-Octacosanol, TMS |  | 0 ± 0 | 0 ± 0 | 0.01 ± 0 | 0.01 ± 0 | 0 ± 0 | 0.01 ± 0 | 0.04 ± 0 | 0 ± 0 | 0.05 ± 0.05 | 0.21 ± 0.01 | 0.13 ± 0.01 | 0.2 ± 0 |
| 17 | 10.22 | 1273 | 2-(*E*)-Decenal |  | 0.05 ± 0.04 | 0.01 ± 0.01 | 0.5 ± 0.03 | 0.32 ± 0.19 | 0.01 ± 0.01 | 0.02 ± 0 | 0.12 ± 0 | 0 ± 0 | 0.03 ± 0.01 | 0.41 ± 0.15 | 0.33 ± 0.13 | 0.46 ± 0.12 |
| Total alcohol/aldehydes | | | |  | **0.37 ± 0.23** | **1.06 ± 0.5** | **26.25 ± 3.72** | **18.85 ± 7.11** | **0.31 ± 0.1** | **0.86 ± 0.23** | **18.09 ± 2.19** | **1.96 ± 2.05** | **1.29 ± 0.73** | **36.49 ± 4.97** | **41.92 ± 0.61** | **52.62 ± 4.96** |
| Amino acid | | | |  |  |  |  |  |  |  |  |  |  |  |  |  |
| 33 | 13.98 | 1535 | Pyroglutamic acid, *N,O*-2TMS |  | 0.01 ± 0.01 | 0.03 ± 0.01 | 0.33 ± 0.06 | 0.11 ± 0.06 | 0.01 ± 0 | 0.04 ± 0.01 | 0.07 ± 0.02 | 0.01 ± 0.01 | 0.02 ± 0.01 | 0.28 ± 0.11 | 0.28 ± 0.11 | 0.35 ± 0.11 |
| 34 | 14.42 | 1568 | L-Threonine, 3TMS |  | 0.01 ± 0.01 | 0.03 ± 0.01 | 0.59 ± 0.06 | 0.19 ± 0.11 | 0.01 ± 0 | 0.04 ± 0 | 0.05 ± 0.01 | 0.01 ± 0 | 0.01 ± 0 | 0.49 ± 0.34 | 0.82 ± 0.61 | 0.65 ± 0.25 |
| Total amino acids | | | |  | **0.02 ± 0.01** | **0.06 ± 0.02** | **0.91 ± 0.12** | **0.3 ± 0.17** | **0.02 ± 0.01** | **0.07 ± 0.01** | **0.12 ± 0.03** | **0.02 ± 0.01** | **0.03 ± 0.01** | **0.77 ± 0.45** | **1.1 ± 0.72** | **1 ± 0.37** |
| Fatty acid/ester | | | |  |  |  |  |  |  |  |  |  |  |  |  |  |
| 7 | 7.16 | 1085 | Caproic acid, TMS |  | 0.03 ± 0.01 | 0.08 ± 0.07 | 4.33 ± 0.05 | 1.34 ± 0.01 | 0.03 ± 0.02 | 0.07 ± 0.03 | 1.42 ± 0.69 | 0.05 ± 0.02 | 0.09 ± 0 | 15.9 ± 14.76 | 16.16 ± 14.49 | 5.56 ± 2.03 |
| 12 | 8.73 | 1178 | Heptanoic acid, TMS |  | 0 ± 0 | 0 ± 0 | 0.23 ± 0.1 | 0.07 ± 0 | 0.01 ± 0 | 0 ± 0 | 0.05 ± 0.04 | 0.01 ± 0 | 0 ± 0 | 1.07 ± 0.74 | 1.41 ± 1.07 | 0.45 ± 0.07 |
| 16 | 10.20 | 1271 | Octanoic acid, TMS |  | 0.06 ± 0.02 | 0.12 ± 0.12 | 1.11 ± 0.4 | 0.37 ± 0.07 | 0.07 ± 0.03 | 0.14 ± 0.01 | 0.56 ± 0.04 | 0.07 ± 0.01 | 0.09 ± 0.02 | 2.37 ± 1.01 | 3.51 ± 1.93 | 1.62 ± 0.13 |
| 25 | 11.60 | 1363 | Nonanoic acid, TMS |  | 0.03 ± 0 | 0.04 ± 0.02 | 0.87 ± 0.14 | 0.38 ± 0.04 | 0.03 ± 0.02 | 0.05 ± 0.01 | 0.3 ± 0.1 | 0.02 ± 0.01 | 0.03 ± 0 | 2.21 ± 1.06 | 1.58 ± 0.18 | 1.01 ± 0.19 |
| 27 | 12.74 | 1441 | Methyl decanoate, TMS |  | 0.01 ± 0.01 | 0.03 ± 0.02 | 2.86 ± 0.88 | 0.79 ± 0.04 | 0.01 ± 0 | 0.02 ± 0 | 1.08 ± 0.09 | 0.02 ± 0 | 0.01 ± 0 | 3.62 ± 0.34 | 4.46 ± 0.01 | 4.04 ± 0.01 |
| 29 | 13.52 | 1500 | Nonanoic acid, 7-oxo-, TMS |  | 0.02 ± 0.01 | 0.05 ± 0.01 | 2.99 ± 0.04 | 1.08 ± 0.47 | 0.01 ± 0.01 | 0.07 ± 0.03 | 0.88 ± 0.09 | 0.03 ± 0.02 | 0.06 ± 0.05 | 3.01 ± 0.91 | 3.16 ± 0.5 | 3.42 ± 0.54 |
| 37 | 15.46 | 1653 | Lauric acid, TMS |  | 0.19 ± 0.13 | 0.23 ± 0.19 | 4.49 ± 1.79 | 1.33 ± 0.37 | 0.22 ± 0.06 | 0.32 ± 0.02 | 2.05 ± 0.37 | 0.28 ± 0.08 | 0.37 ± 0.05 | 4.24 ± 0.82 | 7.31 ± 1.9 | 4.68 ± 0.22 |
| 39 | 16.06 | 1703 | Octanedioic acid, 2TMS |  | 0.07 ± 0.01 | 0.12 ± 0.09 | 1.44 ± 0.48 | 0.37 ± 0.06 | 0.06 ± 0.01 | 0.11 ± 0.07 | 0.38 ± 0.06 | 0.06 ± 0.01 | 0.1 ± 0.04 | 0.87 ± 0.13 | 0.9 ± 0.11 | 0.97 ± 0.03 |
| 42 | 17.18 | 1798 | Nonanedioic acid, 2TMS |  | 0.12 ± 0.02 | 0.17 ± 0.16 | 2.76 ± 0.85 | 0.75 ± 0.07 | 0.11 ± 0.04 | 0.2 ± 0.09 | 0.8 ± 0.09 | 0.1 ± 0.01 | 0.17 ± 0.1 | 1.87 ± 0.02 | 1.99 ± 0.09 | 1.79 ± 0.02 |
| 48 | 17.71 | 1848 | Myristic acid, TMS* |  | 0.12 ± 0.07 | 0.1 ± 0.08 | 4.5 ± 3.09 | 0.86 ± 0.1 | 0.05 ± 0.01 | 0.11 ± 0.01 | 1.18 ± 0.73 | 0.06 ± 0.01 | 0.12 ± 0.02 | 2.77 ± 0.78 | 8.21 ± 5.67 | 3.03 ± 0.12 |
| 52 | 18.24 | 1897 | Sebacic acid, 2TMS |  | 0.01 ± 0.01 | 0.01 ± 0.01 | 0.29 ± 0.14 | 0.06 ± 0.01 | 0.01 ± 0 | 0.01 ± 0 | 0.09 ± 0 | 0.01 ± 0 | 0.01 ± 0 | 0.18 ± 0.01 | 0.24 ± 0.06 | 0.15 ± 0.01 |
| 58 | 18.73 | 1944 | Pentadecanoic acid, TMS |  | 0.01 ± 0 | 0.02 ± 0.01 | 0.44 ± 0.13 | 0.18 ± 0.03 | 0.01 ± 0 | 0.04 ± 0.02 | 0.35 ± 0.04 | 0.01 ± 0 | 0.03 ± 0.03 | 0.64 ± 0.05 | 0.89 ± 0.26 | 0.55 ± 0.01 |
| 60 | 19.24 | 1992 | Undecanedioic acid, 2TMS |  | 0 ± 0 | 0.01 ± 0 | 0.11 ± 0.04 | 0.05 ± 0.02 | 0 ± 0 | 0.01 ± 0 | 0.06 ± 0.03 | 0 ± 0 | 0.01 ± 0 | 0.2 ± 0.02 | 0.13 ± 0.01 | 0.15 ± 0.02 |
| 63 | 19.55 | 2024 | 9-Hexadecenoic acid, TMS |  | 0.01 ± 0.01 | 0.01 ± 0 | 0.21 ± 0.12 | 0.1 ± 0.01 | 0.01 ± 0 | 0.01 ± 0 | 0.13 ± 0.04 | 0.01 ± 0 | 0.01 ± 0 | 0.27 ± 0.03 | 0.43 ± 0.2 | 0.22 ± 0.03 |
| 65 | 19.76 | 2045 | Palmitic acid, TMS |  | 0.85 ± 0.77 | 0.45 ± 0.32 | 53.04 ± 44.43 | 5.6 ± 1.35 | 0.34 ± 0.17 | 0.8 ± 0.45 | 25.78 ± 16.88 | 0.24 ± 0.04 | 0.97 ± 0.71 | 22.67 ± 6.46 | 77.99 ± 58.89 | 19.08 ± 0.4 |
| 67 | 20.72 | 2143 | Heptadecanoic acid, TMS |  | 0.01 ± 0.01 | 0.02 ± 0.01 | 0.41 ± 0.25 | 0.11 ± 0.03 | 0.01 ± 0 | 0.02 ± 0.01 | 0.23 ± 0.05 | 0.01 ± 0 | 0.02 ± 0.01 | 0.39 ± 0.02 | 0.71 ± 0.34 | 0.37 ± 0.03 |
| 68 | 21.43 | 2218 | Linoleic acid, TMS* |  | 0.77 ± 0.51 | 0.71 ± 0.51 | 46.55 ± 35.49 | 6.14 ± 1.42 | 0.57 ± 0.2 | 2.28 ± 1.24 | 28.6 ± 15.1 | 0.67 ± 0.1 | 1.75 ± 1.11 | 15.36 ± 1.07 | 35.3 ± 18.43 | 17.99 ± 2.01 |
| 69 | 21.47 | 2223 | Oleic acid, TMS* |  | 0 ± 0 | 0.01 ± 0 | 0.45 ± 0.25 | 0.09 ± 0.04 | 0 ± 0 | 0.01 ± 0 | 0.19 ± 0.03 | 0.01 ± 0 | 0.01 ± 0 | 0.08 ± 0.02 | 0.13 ± 0.03 | 0.1 ± 0.06 |
| 70 | 21.64 | 2242 | Stearic acid, TMS |  | 0.21 ± 0.04 | 0.3 ± 0.21 | 20.85 ± 15.51 | 3.26 ± 0.56 | 0.2 ± 0.05 | 0.49 ± 0.3 | 11.13 ± 5.6 | 0.12 ± 0.01 | 0.5 ± 0.36 | 13.23 ± 3.39 | 28.43 ± 16.74 | 11.11 ± 0.48 |
| 72 | 23.01 | 2396 | 1-Monostearin, 2TMS derivative |  | 0.02 ± 0.02 | 0.02 ± 0.01 | 0.66 ± 0.41 | 0.19 ± 0.1 | 0.01 ± 0 | 0.02 ± 0.01 | 0.11 ± 0.05 | 0.01 ± 0 | 0.02 ± 0.01 | 0.55 ± 0.31 | 0.68 ± 0.36 | 0.55 ± 0.16 |
| 73 | 23.04 | 2400 | n-Tetradecanoic acid, pentamethyl, 2TMS |  | 0.02 ± 0.01 | 0.04 ± 0.03 | 1.7 ± 1.02 | 1.27 ± 0.53 | 0.01 ± 0 | 0.06 ± 0.01 | 2.03 ± 0.7 | 0.01 ± 0.01 | 0.08 ± 0.04 | 2.02 ± 0.08 | 2.21 ± 1.27 | 2.24 ± 0.14 |
| 74 | 23.17 | 2416 | Eicosadienoic acid, TMS |  | 0.04 ± 0.01 | 0.06 ± 0.04 | 4.27 ± 3.44 | 0.64 ± 0.14 | 0.03 ± 0.01 | 0.11 ± 0.1 | 2.06 ± 1.47 | 0.04 ± 0.01 | 0.12 ± 0.1 | 1.3 ± 0.26 | 2.73 ± 1.61 | 1.64 ± 0.02 |
| 75 | 23.37 | 2441 | Eicosanoic acid, TMS |  | 0.02 ± 0.01 | 0.05 ± 0.03 | 1.93 ± 0.94 | 0.74 ± 0.23 | 0.01 ± 0 | 0.1 ± 0.06 | 1.48 ± 0.23 | 0.02 ± 0 | 0.11 ± 0.09 | 2.91 ± 0.22 | 3.94 ± 0.96 | 2.98 ± 0.1 |
| 76 | 24.64 | 2602 | 1-Monopalmitin, TMS |  | 9.13 ± 5.88 | 10.21 ± 9.06 | 121.1 ± 35.22 | 154.25 ± 59.21 | 9.32 ± 3.44 | 18.04 ± 1.99 | 190.66 ± 27.96 | 8.02 ± 6.52 | 23.66 ± 8.37 | 135.54 ± 1.05 | 121.25 ± 31.48 | 157.08 ± 1.36 |
| 77 | 24.81 | 2623 | Docosadienoic acid, TMS |  | 0.01 ± 0 | 0.01 ± 0.01 | 0.15 ± 0.08 | 0.01 ± 0.01 | 0.01 ± 0.01 | 0.01 ± 0 | 0.04 ± 0.01 | 0.01 ± 0 | 0.01 ± 0.01 | 0.04 ± 0.01 | 0.11 ± 0.03 | 0.07 ± 0 |
| 78 | 24.97 | 2644 | Docosanoic acid, TMS |  | 0.02 ± 0.01 | 0.03 ± 0.02 | 0.76 ± 0.34 | 0.36 ± 0.09 | 0.02 ± 0 | 0.07 ± 0.05 | 0.48 ± 0.04 | 0.01 ± 0 | 0.08 ± 0.05 | 0.88 ± 0 | 1.12 ± 0.19 | 0.97 ± 0.01 |
| 80 | 25.42 | 2701 | Monoolein TMS |  | 0.01 ± 0 | 0.03 ± 0.02 | 10.42 ± 0.47 | 0.08 ± 0.02 | 0 ± 0 | 0.01 ± 0 | 2.54 ± 2.26 | 0.01 ± 0 | 0.01 ± 0.01 | 9.8 ± 9.64 | 0.16 ± 0.09 | 0.09 ± 0.01 |
| 81 | 25.67 | 2733 | 2-Linoleoylglycerol, 2TMS |  | 0.01 ± 0.01 | 0.02 ± 0.02 | 0.64 ± 0.47 | 0.27 ± 0.16 | 0.01 ± 0 | 0.04 ± 0.02 | 0.36 ± 0.12 | 0.01 ± 0 | 0.09 ± 0.07 | 1.11 ± 0.38 | 1.39 ± 0.56 | 0.9 ± 0.17 |
| 82 | 25.72 | 2739 | Tetracosanoic acid, TMS |  | 0 ± 0 | 0.01 ± 0.01 | 0.39 ± 0.28 | 0.13 ± 0.03 | 0 ± 0 | 0.02 ± 0.01 | 0.19 ± 0.01 | 0 ± 0 | 0.04 ± 0.03 | 0.39 ± 0.01 | 0.43 ± 0.03 | 0.38 ± 0.02 |
| 83 | 26.12 | 2789 | Monostearin, 2TMS |  | 13.73 ± 10.02 | 19.19 ± 18.65 | 139.93 ± 35.3 | 214.23 ± 89.96 | 11.66 ± 4.02 | 30.73 ± 4.55 | 220.11 ± 26.93 | 9.5 ± 6.1 | 44.59 ± 20.97 | 151.11 ± 2.32 | 137.98 ± 24.19 | 173.43 ± 1.06 |
| 84 | 26.44 | 2821 | Monoolein, TMS isomer |  | 0.01 ± 0 | 0.02 ± 0.01 | 4.23 ± 3.71 | 0.44 ± 0.2 | 0.01 ± 0.01 | 0.07 ± 0.05 | 0.69 ± 0.07 | 0.01 ± 0 | 0.15 ± 0.13 | 0.86 ± 0.2 | 1.34 ± 0.7 | 0.73 ± 0.2 |
| 87 | 27.47 | 2914 | Glyceryl arachidate, 2(*O*-TMS) |  | 0.01 ± 0 | 0.03 ± 0.04 | 1.07 ± 0.56 | 1.35 ± 0.72 | 0.01 ± 0 | 0.17 ± 0.07 | 1.35 ± 0.28 | 0.02 ± 0.01 | 0.55 ± 0.47 | 1.73 ± 0.01 | 1.52 ± 0.39 | 1.95 ± 0 |
| 89 | 27.85 | 2948 | Hexacosanoic acid, TMS |  | 0.01 ± 0 | 0.01 ± 0.01 | 0.19 ± 0.04 | 0.19 ± 0.04 | 0.01 ± 0 | 0.05 ± 0.03 | 0.21 ± 0 | 0.01 ± 0 | 0.09 ± 0.08 | 0.37 ± 0.01 | 0.32 ± 0 | 0.3 ± 0 |
| 92 | 28.75 | 3030 | Lauric acid glyceryl ester, TMS |  | 0.01 ± 0 | 0.03 ± 0.03 | 0.69 ± 0.2 | 0.76 ± 0.01 | 0.01 ± 0 | 0.11 ± 0.05 | 0.8 ± 0.11 | 0.01 ± 0.01 | 0.49 ± 0.43 | 1.52 ± 0.2 | 1.45 ± 0.22 | 1.52 ± 0.03 |
| 93 | 30.12 | 3153 | 1,2-Dipalmitin, TMS |  | 0.01 ± 0 | 0.03 ± 0.03 | 0.71 ± 0.25 | 0.76 ± 0.02 | 0.01 ± 0 | 0.1 ± 0.03 | 0.86 ± 0.18 | 0.01 ± 0.01 | 0.4 ± 0.35 | 1.41 ± 0.15 | 1.47 ± 0.27 | 1.43 ± 0.02 |
| 94 | 30.46 | 3184 | 1-Laurin-3-Myristin, *O*-TMS |  | 0.01 ± 0.01 | 0.02 ± 0.02 | 0.4 ± 0.08 | 0.47 ± 0.03 | 0.01 ± 0 | 0.05 ± 0.02 | 0.51 ± 0.08 | 0.01 ± 0.01 | 0.28 ± 0.25 | 0.9 ± 0.06 | 0.87 ± 0.12 | 0.93 ± 0.02 |
| 96 | 30.96 | 3228 | 1,3-Dipalmitin, TMS |  | 0.01 ± 0 | 0.02 ± 0.03 | 2.79 ± 2.59 | 0.37 ± 0.02 | 0.01 ± 0.01 | 0.04 ± 0.01 | 0.55 ± 0.04 | 0.01 ± 0.02 | 0.15 ± 0.1 | 0.67 ± 0.02 | 0.63 ± 0.04 | 0.72 ± 0.01 |
| 99 | 31.33 | 3262 | 1,3-Dipalmitin, TMS isomer |  | 0.04 ± 0.05 | 0.19 ± 0.3 | 3.5 ± 0.12 | 9.54 ± 5.12 | 0.02 ± 0.02 | 0.63 ± 0.11 | 8.19 ± 1.51 | 0.03 ± 0.02 | 1.76 ± 1.43 | 8.52 ± 1.51 | 6.88 ± 0.85 | 6.93 ± 1.35 |
| Total fatty acid/esters | | | |  | **25.65 ± 17.66** | **32.49 ± 30.18** | **443.45 ± 189.68** | **408.98 ± 161.34** | **22.93 ± 8.15** | **55.2 ± 9.49** | **508.43 ± 102.12** | **19.52 ± 13.07** | **77.06 ± 35.53** | **412.57 ± 48.09** | **479.42 ± 184.29** | **431.13 ± 11.06** |
| Hydrocarbon | | | |  |  |  |  |  |  |  |  |  |  |  |  |  |
| 85 | 26.88 | 2861 | Octacosane |  | 0.09 ± 0.03 | 0.08 ± 0.07 | 1.43 ± 0.82 | 0.74 ± 0.02 | 0.03 ± 0.01 | 0.4 ± 0.17 | 0.56 ± 0.03 | 0.02 ± 0 | 0.29 ± 0.19 | 1.36 ± 0.22 | 0.77 ± 0.05 | 1.15 ± 0.39 |
| 90 | 28.31 | 2989 | Hentriacontane |  | 0.04 ± 0.02 | 0.06 ± 0.06 | 0.47 ± 0.23 | 0.38 ± 0.08 | 0.02 ± 0.01 | 0.22 ± 0.06 | 0.25 ± 0 | 0.02 ± 0.01 | 0.3 ± 0.19 | 0.53 ± 0.03 | 0.62 ± 0.09 | 0.87 ± 0.03 |
| Total hydrocarbons | | | |  | **0.13 ± 0.05** | **0.14 ± 0.13** | **1.9 ± 1.05** | **1.12 ± 0.1** | **0.05 ± 0.01** | **0.62 ± 0.23** | **0.81 ± 0.03** | **0.04 ± 0.01** | **0.59 ± 0.38** | **1.89 ± 0.25** | **1.39 ± 0.13** | **2.02 ± 0.41** |
| Organic acid | | | |  |  |  |  |  |  |  |  |  |  |  |  |  |
| 1 | 5.98 | 978 | Lactic acid, 2TMS* |  | 0 ± 0 | 0.01 ± 0 | 0.21 ± 0.01 | 0.19 ± 0.08 | 0 ± 0 | 0.01 ± 0 | 0.43 ± 0.1 | 0 ± 0 | 0.01 ± 0.01 | 0.36 ± 0.05 | 0.37 ± 0.01 | 0.31 ± 0.01 |
| 2 | 6.24 | 1001 | Tiglic acid, TMS |  | 0 ± 0 | 0 ± 0 | 14.64 ± 1.97 | 11.98 ± 3.54 | 0 ± 0 | 0 ± 0 | 17.39 ± 1.96 | 0 ± 0 | 0.01 ± 0 | 25.41 ± 1.29 | 27.11 ± 0.08 | 27.29 ± 0.11 |
| 5 | 7.06 | 1076 | Butanoic acid, 2-hydroxy, 2TMS |  | 0 ± 0 | 0.01 ± 0 | 0.25 ± 0.01 | 0.17 ± 0.05 | 0 ± 0.01 | 0.01 ± 0 | 0.36 ± 0.07 | 0 ± 0 | 0.01 ± 0.01 | 0.5 ± 0.05 | 0.42 ± 0.03 | 0.45 ± 0.03 |
| 6 | 7.10 | 1080 | Glycolic acid, 2TMS |  | 0.01 ± 0 | 0.05 ± 0.05 | 1.49 ± 0.04 | 0.7 ± 0.64 | 0.01 ± 0.01 | 0.01 ± 0 | 3.83 ± 0.9 | 0.02 ± 0.01 | 0.07 ± 0.06 | 2.93 ± 0.93 | 3.1 ± 0.22 | 3.45 ± 0.71 |
| 8 | 7.22 | 1091 | Pyruvic acid, 2TMS |  | 0 ± 0 | 0.04 ± 0.06 | 0.01 ± 0.01 | 0.51 ± 0.5 | 0.01 ± 0 | 0.01 ± 0 | 0.01 ± 0.01 | 0.01 ± 0 | 0.04 ± 0.03 | 0.01 ± 0 | 1.63 ± 1.62 | 0.01 ± 0 |
| 9 | 7.78 | 1125 | Malonic acid, 2TMS |  | 0 ± 0 | 0.01 ± 0.01 | 1.43 ± 0.18 | 0.49 ± 0.46 | 0.01 ± 0 | 0.01 ± 0 | 0.93 ± 0.08 | 0.01 ± 0.01 | 0.02 ± 0 | 2.93 ± 0.27 | 2.58 ± 0.05 | 2.69 ± 0.05 |
| 10 | 8.40 | 1160 | 3-Hydroxypropionic acid, 2TMS |  | 0 ± 0 | 0.01 ± 0.01 | 0.13 ± 0.01 | 0.11 ± 0.04 | 0 ± 0 | 0.01 ± 0.01 | 0.1 ± 0.01 | 0.01 ± 0 | 0.01 ± 0 | 0.39 ± 0.09 | 0.39 ± 0.08 | 0.36 ± 0.04 |
| 13 | 9.10 | 1199 | 3-Hydroxyisovaleric acid, 2TMS |  | 0 ± 0 | 0 ± 0 | 0.11 ± 0.02 | 0.07 ± 0.01 | 0 ± 0 | 0.01 ± 0 | 0.06 ± 0 | 0 ± 0 | 0 ± 0 | 0.19 ± 0.01 | 0.2 ± 0.01 | 0.19 ± 0 |
| 14 | 9.81 | 1245 | 4-Hydroxybutyric acid, 2TMS |  | 0 ± 0 | 0.01 ± 0.01 | 0.23 ± 0.07 | 0.09 ± 0.02 | 0 ± 0 | 0.02 ± 0.01 | 0.11 ± 0 | 0.01 ± 0 | 0.02 ± 0 | 0.48 ± 0.02 | 0.49 ± 0.05 | 0.5 ± 0.01 |
| 15 | 10.00 | 1257 | Benzoic acid, TMS* |  | 0 ± 0 | 0 ± 0 | 0.31 ± 0.08 | 0.11 ± 0.04 | 0 ± 0 | 0 ± 0 | 0.15 ± 0.02 | 0 ± 0 | 0 ± 0 | 0.63 ± 0.07 | 0.63 ± 0.02 | 0.58 ± 0 |
| 20 | 10.79 | 1309 | Phenylacetic acid, TMS |  | 0 ± 0 | 0.01 ± 0 | 1.27 ± 0.69 | 0.25 ± 0.23 | 0 ± 0 | 0.02 ± 0 | 0.61 ± 0.26 | 0 ± 0 | 0.01 ± 0 | 0.08 ± 0.06 | 0.16 ± 0.11 | 0.27 ± 0.37 |
| 21 | 10.99 | 1321 | Succinic acid, 2TMS |  | 0.01 ± 0 | 0.01 ± 0.01 | 0.15 ± 0.02 | 0.06 ± 0.03 | 0 ± 0 | 0.01 ± 0 | 0.06 ± 0.01 | 0.01 ± 0 | 0.01 ± 0 | 0.21 ± 0.01 | 0.24 ± 0.06 | 0.36 ± 0.06 |
| 22 | 11.17 | 1333 | Methyl succinic acid, 2TMS |  | 0.01 ± 0 | 0.06 ± 0.05 | 0.48 ± 0.13 | 0.18 ± 0.03 | 0.02 ± 0.01 | 0.08 ± 0.03 | 0.26 ± 0.04 | 0.02 ± 0.01 | 0.09 ± 0.06 | 1.38 ± 0.06 | 1.68 ± 0.12 | 1.68 ± 0.06 |
| 23 | 11.31 | 1343 | Methyl maleic acid, 2TMS |  | 0 ± 0 | 0.01 ± 0 | 0.04 ± 0 | 0.02 ± 0.01 | 0 ± 0 | 0.01 ± 0 | 0.03 ± 0.01 | 0 ± 0 | 0.01 ± 0 | 0.09 ± 0.08 | 0.11 ± 0 | 0.11 ± 0.01 |
| 24 | 11.44 | 1351 | Fumaric acid, 2TMS |  | 0 ± 0 | 0 ± 0 | 0.03 ± 0 | 0.01 ± 0 | 0 ± 0 | 0 ± 0 | 0.01 ± 0 | 0 ± 0 | 0 ± 0 | 0.04 ± 0.02 | 0.05 ± 0.01 | 0.03 ± 0.01 |
| 28 | 13.42 | 1492 | Unknown organic acid |  | 0.01 ± 0 | 0.01 ± 0.01 | 0.26 ± 0.07 | 0.1 ± 0 | 0.01 ± 0 | 0.01 ± 0 | 0.11 ± 0 | 0.01 ± 0 | 0.01 ± 0 | 0.35 ± 0.06 | 0.4 ± 0 | 0.35 ± 0.02 |
| 31 | 13.77 | 1520 | Methyl maleic acid, 2TMS |  | 0 ± 0 | 0 ± 0 | 0.65 ± 0.18 | 0.13 ± 0 | 0 ± 0 | 0 ± 0 | 0.15 ± 0.01 | 0 ± 0 | 0 ± 0 | 0.85 ± 0.05 | 1.05 ± 0.07 | 0.98 ± 0.02 |
| 35 | 15.09 | 1621 | 5-Hydroxypipecolic acid, 3TMS |  | 0 ± 0 | 0 ± 0 | 0.02 ± 0 | 0.01 ± 0.01 | 0 ± 0 | 0.01 ± 0 | 0.01 ± 0.01 | 0 ± 0 | 0.01 ± 0.01 | 0.01 ± 0 | 0.02 ± 0.01 | 0.01 ± 0.01 |
| Total organic acids | | | |  | **0.07 ± 0.02** | **0.26 ± 0.22** | **21.71 ± 3.5** | **15.18 ± 5.69** | **0.09 ± 0.04** | **0.24 ± 0.06** | **24.61 ± 3.5** | **0.11 ± 0.05** | **0.33 ± 0.2** | **36.84 ± 3.12** | **40.66 ± 2.55** | **39.62 ± 1.51** |
| Steroid/terpenoid | | | |  |  |  |  |  |  |  |  |  |  |  |  |  |
| 26 | 12.22 | 1402 | (-)-β-Elemene |  | 0.01 ± 0 | 0.01 ± 0 | 5.04 ± 5.02 | 0.01 ± 0 | 0 ± 0 | 0.01 ± 0 | 0.94 ± 0.92 | 0.01 ± 0 | 0.01 ± 0 | 0.04 ± 0.02 | 0.03 ± 0.02 | 0.05 ± 0.01 |
| 30 | 13.71 | 1515 | β-Caryophyllene* |  | 0.01 ± 0 | 0.01 ± 0 | 0.64 ± 0.63 | 0.01 ± 0 | 0.01 ± 0 | 0.01 ± 0 | 0.17 ± 0.15 | 0.01 ± 0 | 0.02 ± 0.01 | 0.07 ± 0.04 | 0.03 ± 0 | 0.06 ± 0.03 |
| 71 | 21.94 | 2276 | Norepiandrosterone, TMS |  | 0.16 ± 0.01 | 0.39 ± 0.36 | 9.44 ± 3.38 | 3.39 ± 0.96 | 0.13 ± 0.04 | 0.62 ± 0.47 | 5.57 ± 0.36 | 0.09 ± 0.01 | 0.69 ± 0.59 | 12.04 ± 0.59 | 15.93 ± 2.78 | 12.74 ± 0.81 |
| 88 | 27.54 | 2921 | Ursolic acid, 2TMS |  | 0.01 ± 0 | 0.01 ± 0 | 1.15 ± 0.62 | 1.19 ± 0.2 | 0.01 ± 0 | 0.02 ± 0.01 | 1.97 ± 0.13 | 0 ± 0 | 0.32 ± 0.31 | 2.22 ± 0.32 | 1.6 ± 0.13 | 1.32 ± 0.01 |
| 98 | 31.09 | 3240 | β-Sitosterol, TMS* |  | 0.03 ± 0.01 | 0.1 ± 0.06 | 3 ± 0.7 | 2.97 ± 0.84 | 0.02 ± 0.01 | 0.31 ± 0.19 | 2.42 ± 0.15 | 0.04 ± 0.02 | 1.59 ± 1.37 | 2.96 ± 0.85 | 2.42 ± 0.07 | 4 ± 0.73 |
| Total steroid/terpenoids | | | |  | **0.2 ± 0.03** | **0.51 ± 0.42** | **19.26 ± 10.35** | **7.58 ± 2** | **0.17 ± 0.05** | **0.96 ± 0.67** | **11.08 ± 1.69** | **0.15 ± 0.04** | **2.63 ± 2.28** | **17.33 ± 1.82** | **20.02 ± 2.99** | **18.17 ± 1.59** |
| Sugar | | | |  |  |  |  |  |  |  |  |  |  |  |  |  |
| 36 | 15.16 | 1627 | Ribofuranose, 4TMS |  | 0 ± 0 | 0 ± 0 | 0.03 ± 0 | 0.01 ± 0 | 0 ± 0 | 0 ± 0 | 0.01 ± 0 | 0 ± 0 | 0 ± 0 | 0.01 ± 0 | 0.05 ± 0.03 | 0.02 ± 0.01 |
| 41 | 16.91 | 1775 | Arabinofuranose, 4TMS |  | 0 ± 0 | 0 ± 0 | 0.03 ± 0.01 | 0.01 ± 0.01 | 0 ± 0 | 0 ± 0 | 0.02 ± 0 | 0 ± 0 | 0 ± 0 | 0.07 ± 0.05 | 0.05 ± 0 | 0.03 ± 0.01 |
| 43 | 17.47 | 1825 | Fructofuranose, 5TMS |  | 0.01 ± 0.01 | 0.07 ± 0.05 | 7.05 ± 2.68 | 3.73 ± 2.88 | 0.01 ± 0 | 0.02 ± 0.01 | 24.89 ± 11.35 | 0.03 ± 0.02 | 0.65 ± 0.63 | 6.48 ± 4.66 | 7.22 ± 5.6 | 3.68 ± 2.24 |
| 44 | 17.49 | 1827 | Mannose, 5TMS |  | 0 ± 0 | 0 ± 0 | 0.13 ± 0.04 | 0.05 ± 0.04 | 0 ± 0 | 0 ± 0 | 0.18 ± 0.07 | 0 ± 0 | 0.01 ± 0 | 0.09 ± 0.06 | 0.19 ± 0.13 | 0.14 ± 0.07 |
| 46 | 17.56 | 1833 | Psicofuranose, 5TMS |  | 0.02 ± 0.02 | 0.1 ± 0.05 | 5.64 ± 1.47 | 2.7 ± 1.94 | 0.02 ± 0.01 | 0.05 ± 0.03 | 18.82 ± 10.22 | 0.07 ± 0.06 | 1.04 ± 0.99 | 5.31 ± 3.36 | 5.11 ± 3.34 | 2.97 ± 1.23 |
| 47 | 17.66 | 1842 | Fructopyranose, 5TMS |  | 0.09 ± 0.04 | 0.04 ± 0.03 | 1.82 ± 0.64 | 0.79 ± 0.57 | 0.04 ± 0 | 0.01 ± 0 | 3.42 ± 1.66 | 0.02 ± 0.03 | 0.05 ± 0 | 1.53 ± 0.89 | 2.11 ± 1.2 | 1.22 ± 0.62 |
| 51 | 17.90 | 1865 | Talofuranose, 5TMS |  | 0.01 ± 0 | 0.02 ± 0.01 | 0.74 ± 0.05 | 0.38 ± 0.26 | 0.01 ± 0 | 0.01 ± 0 | 2.03 ± 0.85 | 0.01 ± 0.01 | 0.15 ± 0.14 | 1.04 ± 0.49 | 0.86 ± 0.22 | 1.41 ± 0.63 |
| 53 | 18.36 | 1908 | Mannonic acid, 1,5-lactone, 4TMS |  | 0 ± 0 | 0 ± 0 | 0.93 ± 0.26 | 0.76 ± 0.61 | 0 ± 0 | 0 ± 0 | 0.9 ± 0.84 | 0 ± 0 | 0.01 ± 0.01 | 0.96 ± 0.56 | 1.07 ± 0.65 | 1.8 ± 0.78 |
| 54 | 18.40 | 1912 | Psicose, 5TMS |  | 0 ± 0 | 0 ± 0 | 0.36 ± 0.09 | 0.16 ± 0.11 | 0 ± 0 | 0 ± 0 | 1.1 ± 0.59 | 0 ± 0 | 0.01 ± 0.01 | 0.41 ± 0.28 | 0.5 ± 0.34 | 0.28 ± 0.17 |
| 55 | 18.45 | 1917 | Mannopyranose, 5TMS |  | 0 ± 0 | 0.03 ± 0.03 | 5.52 ± 1.62 | 4.22 ± 3.35 | 0 ± 0 | 0.01 ± 0 | 21.56 ± 9.81 | 0.01 ± 0 | 0.07 ± 0.06 | 6.63 ± 3.98 | 7 ± 4.34 | 14.89 ± 6.21 |
| 56 | 18.50 | 1922 | Gluconic acid, 1,4-lactone, 4TMS |  | 0 ± 0 | 0.01 ± 0.01 | 0.48 ± 0.39 | 1.05 ± 0.83 | 0 ± 0 | 0 ± 0 | 5.91 ± 2.82 | 0.01 ± 0.01 | 0.02 ± 0.01 | 1.78 ± 1.11 | 1.56 ± 1.54 | 3.27 ± 1.49 |
| 57 | 18.57 | 1929 | D-Glucose, 5TMS |  | 0 ± 0 | 0.01 ± 0 | 0.25 ± 0.01 | 0.11 ± 0.08 | 0 ± 0 | 0 ± 0 | 0.23 ± 0.03 | 0 ± 0 | 0.03 ± 0.02 | 0.19 ± 0.08 | 0.41 ± 0.18 | 0.37 ± 0.19 |
| 61 | 19.34 | 2002 | Glucopyranose, 5TMS* |  | 0 ± 0 | 0.04 ± 0.05 | 9.09 ± 3.58 | 9.38 ± 7.79 | 0 ± 0 | 0.01 ± 0 | 37.59 ± 16.52 | 0.01 ± 0 | 0.08 ± 0.07 | 11.54 ± 7.62 | 11.68 ± 8.31 | 23.4 ± 8.92 |
| 64 | 19.63 | 2031 | Gluconic acid, 6TMS |  | 0.01 ± 0.01 | 0.01 ± 0 | 2.25 ± 0.89 | 0.66 ± 0.35 | 0 ± 0 | 0.01 ± 0.01 | 0.69 ± 0.35 | 0.03 ± 0.02 | 0.02 ± 0.01 | 0.25 ± 0.09 | 1.23 ± 0.98 | 0.52 ± 0.29 |
| 79 | 25.34 | 2690 | Sucrose, 8TMS* |  | 0 ± 0 | 0.02 ± 0.01 | 11.39 ± 5.75 | 14.39 ± 11.15 | 0 ± 0 | 0.09 ± 0.05 | 10.92 ± 6.66 | 0 ± 0 | 0.41 ± 0.4 | 33.39 ± 17.65 | 18.53 ± 14.35 | 14.43 ± 7.97 |
| 95 | 30.80 | 3214 | Mannobiose, 8TMS |  | 0 ± 0 | 0 ± 0 | 0.03 ± 0.01 | 0.09 ± 0.05 | 0 ± 0 | 0.01 ± 0 | 0.03 ± 0.01 | 0 ± 0 | 0.01 ± 0 | 0.05 ± 0.03 | 0.01 ± 0 | 0.09 ± 0.05 |
| 97 | 31.05 | 3237 | Trehalose, 8TMS |  | 0 ± 0 | 0 ± 0 | 0.19 ± 0.05 | 0.39 ± 0.25 | 0 ± 0 | 0.03 ± 0.02 | 0.18 ± 0.02 | 0 ± 0 | 0.03 ± 0.03 | 0.4 ± 0.27 | 0.06 ± 0.03 | 0.95 ± 0.48 |
| 101 | 32.45 | 3363 | Maltose, 8TMS |  | 0 ± 0 | 0 ± 0 | 0.37 ± 0.19 | 1.69 ± 1.13 | 0 ± 0 | 0.02 ± 0.01 | 0.41 ± 0.28 | 0 ± 0 | 0.02 ± 0.02 | 0.66 ± 0.55 | 0.11 ± 0.07 | 1.28 ± 0.96 |
| Total sugars | | | |  | **0.16 ± 0.09** | **0.38 ± 0.26** | **46.29 ± 17.72** | **40.57 ± 31.41** | **0.1 ± 0.03** | **0.27 ± 0.15** | **128.88 ± 62.07** | **0.21 ± 0.17** | **2.61 ± 2.43** | **70.80 ± 41.74** | **57.76 ± 41.3** | **70.77 ± 32.32** |
| Sugar alcohol | | | |  |  |  |  |  |  |  |  |  |  |  |  |  |
| 32 | 13.82 | 1523 | L-Threitol, 4TMS |  | 0 ± 0 | 0 ± 0 | 0.05 ± 0.02 | 0.01 ± 0 | 0 ± 0 | 0 ± 0 | 0.02 ± 0 | 0 ± 0 | 0 ± 0 | 0.07 ± 0.01 | 0.08 ± 0 | 0.07 ± 0 |
| 40 | 16.50 | 1740 | Ribitol, 5TMS |  | 0.01 ± 0 | 0.02 ± 0 | 0.43 ± 0.05 | 0.28 ± 0.19 | 0.01 ± 0 | 0.01 ± 0 | 0.27 ± 0.08 | 0.02 ± 0.01 | 0.03 ± 0.02 | 0.36 ± 0.25 | 0.52 ± 0.23 | 1.3 ± 0.67 |
| 50 | 17.81 | 1857 | D-Pinitol, 5TMS |  | 0.02 ± 0.01 | 0.01 ± 0.01 | 0.14 ± 0 | 0.05 ± 0.03 | 0 ± 0 | 0.06 ± 0.01 | 0.01 ± 0 | 0 ± 0 | 0.01 ± 0 | 0.11 ± 0.06 | 0.08 ± 0.01 | 0.17 ± 0.11 |
| 59 | 18.86 | 1956 | Mannitol, 6TMS |  | 0.01 ± 0 | 0.02 ± 0.01 | 1.65 ± 0.73 | 1.22 ± 1.04 | 0 ± 0 | 0.02 ± 0.01 | 0.53 ± 0.33 | 0 ± 0 | 0.02 ± 0.01 | 0.97 ± 0.72 | 1.15 ± 0.92 | 2.45 ± 1.89 |
| 62 | 19.44 | 2012 | Myo-inositol, 6TMS |  | 0 ± 0 | 0.01 ± 0.01 | 0.04 ± 0.01 | 0.01 ± 0 | 0 ± 0 | 0.01 ± 0 | 24.78 ± 24.78 | 0 ± 0 | 0.01 ± 0 | 0.05 ± 0.01 | 0.01 ± 0 | 0.02 ± 0.01 |
| 66 | 20.50 | 2120 | Myo-inositol, 6TMS isomer |  | 0 ± 0 | 0.01 ± 0.01 | 0.43 ± 0.12 | 0.32 ± 0.19 | 0 ± 0 | 0 ± 0 | 0.37 ± 0.22 | 0 ± 0 | 0.02 ± 0.01 | 0.69 ± 0.45 | 0.44 ± 0.2 | 0.43 ± 0.27 |
| 100 | 31.72 | 3297 | Maltitol, 9TMS |  | 0 ± 0 | 0 ± 0 | 0.39 ± 0.07 | 0.21 ± 0.12 | 0 ± 0 | 0 ± 0 | 0.68 ± 0.16 | 0 ± 0 | 0.02 ± 0.01 | 0.84 ± 0.59 | 0.38 ± 0.27 | 0.34 ± 0.04 |
|  |  |  | **Total sugar alcohols** |  | **0.04 ± 0.02** | **0.07 ± 0.04** | **3.14 ± 1.00** | **2.1 ± 1.58** | **0.02 ± 0.01** | **0.1 ± 0.02** | **26.67 ± 25.56** | **0.04 ± 0.02** | **0.1 ± 0.06** | **3.07 ± 2.09** | **2.67 ± 1.64** | **4.72 ± 2.99** |

* Metabolites with asterisk are confirmed by comparison with standards and quantification for sugars, amino acid, organic acids, and fatty acids were made using glucose, glycine, lactic acid, and oleic acid, respectively.

**Table S2.** Silylated flavonoids and phenolic acids in date palm (*P. dactylifera* L.) seeds expressed as relative percentile analyzed using GC–MS represented as mean ± standard deviation (n = 3)

| Peak no. | | RT (min) | | RI | | Metabolite name |  | Barhi | Omeldehn | Rothana | Lolo | Nabout Seif | Zamli | Khalas | Farsi | Hasawi | Zaghloul | Aref | Breem |
| --- | --- | --- | --- | --- | --- | --- | --- | --- | --- | --- | --- | --- | --- | --- | --- | --- | --- | --- | --- |
| Flavonoids | | | | | | |  |  |  |  |  |  |  |  |  |  |  |  |  |
| 86 | 26.99 | | 2871 | | Catechin, 5TMS | |  | 0.04 ± 0.03 | 0.08 ± 0.03 | 0.69 ± 0.29 | 0.62 ± 0.43 | 0.04 ± 0.01 | 0.15 ± 0.08 | 0.77 ± 0.2 | 0.09 ± 0.01 | 1.48 ± 1.19 | 1.56 ± 0.83 | 0.68 ± 0.36 | 0.67 ± 0.17 |
| Total flavonoids | | | | | | |  | **0.04 ± 0.03** | **0.08 ± 0.03** | **0.69 ± 0.29** | **0.62 ± 0.43** | **0.04 ± 0.01** | **0.15 ± 0.08** | **0.77 ± 0.2** | **0.09 ± 0.01** | **1.48 ± 1.19** | **1.56 ± 0.83** | **0.68 ± 0.36** | **0.67 ± 0.17** |
| Phenolic acids | | | | | | |  |  |  |  |  |  |  |  |  |  |  |  |  |
| 38 | 15.73 | | 1675 | | 4-Methoxymandelic acid, TMS | |  | 0.08 ± 0.05 | 0.18 ± 0.13 | 0.04 ± 0.01 | 0.01 ± 0.01 | 0.02 ± 0.01 | 0.17 ± 0.05 | 0 ± 0 | 0.04 ± 0.01 | 0.03 ± 0.02 | 0.03 ± 0.01 | 0.01 ± 0.01 | 0.03 ± 0 |
| 45 | 17.52 | | 1830 | | Protocatechuic acid, TMS | |  | 0.04 ± 0.02 | 0.09 ± 0.12 | 0.05 ± 0.02 | 0.02 ± 0.01 | 0.04 ± 0.02 | 0.02 ± 0 | 0.03 ± 0.01 | 0.04 ± 0.01 | 0.02 ± 0.01 | 0.04 ± 0.01 | 0.05 ± 0.02 | 0.04 ± 0 |
| 49 | 17.78 | | 1855 | | Sinapic acid, 2TMS | |  | 0.06 ± 0.02 | 0.05 ± 0.02 | 0.06 ± 0.01 | 0.03 ± 0.01 | 0.04 ± 0.01 | 0.03 ± 0.01 | 0.02 ± 0 | 0.05 ± 0.02 | 0.03 ± 0 | 0.08 ± 0.01 | 0.09 ± 0.02 | 0.06 ± 0.01 |
| Total phenolic acids | | | | | | |  | **0.18 ± 0.09** | **0.32 ± 0.27** | **0.15 ± 0.03** | **0.06 ± 0.02** | **0.1 ± 0.04** | **0.21 ± 0.06** | **0.05 ± 0.01** | **0.12 ± 0.05** | **0.08 ± 0.03** | **0.15 ± 0.03** | **0.15 ± 0.04** | **0.13 ± 0.02** |

**Table S3**: List of identified volatile metabolites by HS-SPME/GC-MS, where the relative abundances of each metabolite and phytochemical class are expressed as mean relative abundance (%) ±SD in palm seed cvs. (n=3)

| **peak** | **Average Rt (min)** | **Metabolite name** | **Class** | **Barhi** | **Om Eldehn** | **Rothana** | **Lolo** | **Nabout Seif** | **Khalas** | **Farsi** | **Breem** |
| --- | --- | --- | --- | --- | --- | --- | --- | --- | --- | --- | --- |
| 1 | 5.349 | Butanoic acid, 2-methyl- | **Acid** | 2.0±0.7 | 1.5±1.2 | 0.8±0.8 | 2.3±1.2 | 0.0±0.0 | 0.0±0.0 | 0.3±0.5 | 1.8±0.7 |
| 2 | 9.036 | Octanoic acid |  | 1.7±0.2 | 1.8±1.4 | 4.8±0.9 | 4.5±1.0 | 2.7±0.4 | 2.1±1.6 | 4.4±0.6 | 3.3±1.0 |
| 3 | 10.848 | Pentadecanoic acid |  | 0.3±0.0 | 1.0±0.4 | 0.9±0.2 | 1.0±0.2 | 0.5±0.1 | 0.4±0.4 | 1.0±0.2 | 0.7±0.3 |
| **Total acids (%)** | | | | **4.1±0.9** | **4.3±3.0** | **6.4±1.9** | **7.8±2.4** | **3.2±0.5** | **2.5±2.0** | **5.6±1.2** | **5.8±2.0** |
| 4 | 6.953 | *α*-Methylstyrol | **Alcohol** | 0.9±0.2 | 0.3±0.0 | 0.4±0.1 | 0.1±0.0 | 0.6±0.2 | 0.03±0.0 | 0.1±0.0 | 0.1±0.0 |
| 5 | 8.148 | *α*-Cumyl alcohol |  | 0.5±0.0 | 0.2±0.0 | 0.2±0.0 | 0.1±0.0 | 0.3±0.1 | 0.03±0.0 | 0.1±0.0 | 0.5±0.0 |
| 6 | 9.101 | *dl*-Menthol* |  | 0.1±0.0 | 0.1±0.0 | 0.1±0.0 | 0.1±0.0 | 0.1±0.0 | 0.3±0.1 | 0.1±0.0 | 0.1±0.0 |
| 7 | 9.167 | 4-Terpineol |  | 0.2±0.0 | 0.0±0.0 | 0.01±0.0 | 0.04±0.0 | 0.01±0.0 | 0.02±0.0 | 0.01±0.0 | 0.02±0. |
| 8 | 13.277 | Isospathulenol |  | 0.1±0.0 | 0.1±0.0 | 0.03±0.0 | 0.03±0.0 | 0.01±0.0 | 0.01±0.0 | 0.02±0.0 | 0.03±0. |
| 9 | 13.794 | Espatulenol |  | 0.1±0.0 | 0.1±0.0 | 0.04±0.0 | 0.04±0.0 | 0.02±0.0 | 0.02±0.0 | 0.02±0.0 | 0.1±0. |
| 10 | 14.032 | *trans*-Sesquisabinene hydrate |  | 0.04±0.0 | 0.04±0.0 | 0.02±0.0 | 0.02±0.0 | 0.01±0.0 | 0.01±0.0 | 0.02±0.0 | 0.03±0.0 |
| **Total alcohols (%)** | | | | **1.9±0.3** | **0.8±0.2** | **0.9±0.2** | **0.4±0.1** | **1.1±0.3** | **0.4±0.1** | **0.4±0.1** | **0.9±0.1** |
| 11 | 9.937 | *p*-Anisaldehyde | **Aldehyde** | 2.8±0.2 | 3.5±0.3 | 1.9±0.0 | 2.4±0.3 | 1.0±0. 2 | 0.7±0.5 | 1.6±0.3 | 2.7±1.0 |
| 12 | 10.105 | (*E*)-Cinnamaldehyde* |  | 1.5±1.2 | 0.8±0.7 | 0.3±0.0 | 2.3±3.2 | 0.6±0.3 | 0.7±0.5 | 0.3±0.1 | 0.9±0.4 |
| **Total aldehydes (%)** | | | | **4.4±1.4** | **4.2±1.0** | **2.2±0.4** | **4.7±3.5** | **1.5±0.6** | **1.4±0.9** | **1.9±0.4** | **3.6±1.4** |
| 14 | 7.195 | *cis*-3-Hexenyl acetate | **Ester** | 4.5±2.2 | 6.0±0.9 | 11.3±1.8 | 5.7±2.3 | 11.0±2.1 | 21.1±7.2 | 10.3±3.5 | 7.5±2.1 |
| 13 | 8.33 | Benzoic acid, methyl ester |  | 0.0±0.0 | 0.0±0.0 | 0.01±0.0 | 0.01±0.0 | 0.01±0.0 | 0.01±0.0 | 0.01±0.0 | 0.01±0.0 |
| 15 | 8.512 | Octanoic acid, methyl ester |  | 9.1±2.0 | 9.0±1.1 | 11.6±1.9 | 7.4±0.6 | 12.4±0.2 | 12.2±2.2 | 9.2±1.6 | 7.1±1.4 |
| 16 | 9.259 | Octanoic acid, ethyl ester |  | 2.4±0.1 | 4.9±0.1 | 4.2±0.6 | 2.7±0.6 | 3.5±0.9 | 3.6±1.0 | 5.5±0.8 | 5.8±0.9 |
| 17 | 9.533 | Nonanoic acid, methyl ester |  | 0.6±0.0 | 0.7±0.1 | 0.6±0.1 | 0.2±0.0 | 0.4±0.2 | 0.7±0.2 | 1.1±0.1 | 0.3±0.0 |
| 18 | 10.491 | Decanoic acid, methyl ester |  | 3.6±0.0 | 4.1±0.2 | 5.1±0.6 | 2.9±0.4 | 4.6±0.7 | 3.6±0.9 | 4.2±0.4 | 2.8±0.4 |
| **Total esters (%)** | | | | **20.1±4.3** | **24.6±2.4** | **32.7±5.0** | **18.8±3.5** | **31.9±4.1** | **41.2±11.5** | **30.4±6.5** | **23.4±4.8** |
| 19 | 11.384 | Undecanoic acid, methyl ester | **Fatty acid/ ester** | 0.1±0.0 | 0.1±0.0 | 0.5±0.6 | 0.0±0.0 | 0.1±0.0 | 0.0±0.0 | 0.1±0.0 | 0.0±0.0 |
| 20 | 12.371 | Dodecanoic acid, methyl ester |  | 12.2±0.1 | 12.7±0.4 | 15.9±1.4 | 12.4±0.3 | 18.0±2.4 | 16.8±0.2 | 14.7±1.2 | 11.7±0.7 |
| 21 | 12.804 | n-Hexadecanoic acid |  | 2.9±0.2 | 2.3±1.7 | 6.3±1.1 | 6.9±0.7 | 4.1±1.0 | 3.0±2.1 | 6.1±0.2 | 4.7±1.5 |
| 22 | 13.161 | Dodecanoic acid, ethyl ester |  | 6.1±0.2 | 9.2±0.4 | 7.7±0.6 | 6.8±0.3 | 8.4±0.0.2 | 6.3±1.4 | 11.7±0.8 | 11.9±0.7 |
| 23 | 13.531 | Tridecanoic acid, methyl ester |  | 0.1±0.0 | 0.1±0.0 | 0.1±0.0 | 0.03±0.0 | 0.1±0.0 | 0.02±0.0 | 0.1±0.0 | 0.0±0.0 |
| 24 | 13.838 | Dodecanoic acid |  | 0.1±0.0 | 0.1±0.0 | 0.1±0.1 | 0.2±0.1 | 0.1±0.0 | 0.3±0.3 | 0.1±0.1 | 0.1±0.0 |
| 25 | 14.322 | Eicosanoic acid, ethyl ester |  | 0.2±0.2 | 0.1±0.1 | 0.0±0.0 | 0.1±0.1 | 0.1±0.1 | 0.4±0.5 | 0.2±0.2 | 0.0±0.1 |
| 26 | 14.44 | Methyl isomyristate |  | 4.2±0.1 | 3.7±0.2 | 4.6±0.3 | 3.4±0.3 | 4.8±0.1 | 3.9±1.2 | 4.5±0.3 | 3.2±0.7 |
| 27 | 14.593 | Isobutyl laurate |  | 0.03±0.0 | 0.1±0.0 | 0.03±0.0 | 0.04±0.0 | 0.03±0.0 | 0.02±0.0 | 0.1±0.0 | 0.1±0.0 |
| 28 | 14.707 | Octadecanoic acid |  | 0.2±0.0 | 0.5±0.1 | 0.6±0.6 | 0.6±0.4 | 0.3±0.1 | 0.4±0.3 | 0.5±0.1 | 0.5±0.1 |
| 29 | 14.983 | Tetradecanoic acid, ethyl ester |  | 1.3±0.2 | 2.1±0.0 | 1.2±0.1 | 1.1±0.3 | 1.3±0.3 | 0.8±0.4 | 2.9±0.2 | 2.9±0.8 |
| 30 | 15.477 | *trans*-Pseudoisoeugenyl 2-methylbutyrate |  | 2.8±0.5 | 1.1±1.2 | 0.5±0.8 | 0.8±0.7 | 0.0±0.0 | 0.2±0.2 | 0.5±0.5 | 0.3±0.5 |
| 31 | 15.841 | Linoleic acid, methyl ester |  | 0.02±0.0 | 0.0±0.0 | 0.0±0.0 | 0.0±0.0 | 0.0±0.0 | 0.0±0.0 | 0.0±0.0 | 0.0±0.0 |
| 32 | 15.935 | Elaidic acid, methyl ester |  | 0.1±0.1 | 0.0±0.0 | 0.01±0.0 | 0.0±0.0 | 0.0±0.0 | 0.0±0.0 | 0.0±0.0 | 0.0±0.0 |
| 33 | 16.16 | Pentadecanoic acid, 14-methyl-, methyl ester |  | 0.6±0.6 | 0.0±0.0 | 0.3±0.5 | 0.0±0.0 | 0.0±0.0 | 0.1±0.2 | 0.4±0.7 | 0.1±0.2 |
| 34 | 16.9 | Hexadecanoic acid, ethyl ester |  | 0.1±0.1 | 0.0±0.0 | 0.04±0. | 0.0±0.0 | 0.0±0.0 | 0.02±0.0 | 0.2±0.3 | 0.1±0.2 |
| 35 | 17.122 | (*E*)-9-Octadecenoic acid ethyl ester |  | 0.0±0.0 | 0.0±0.0 | 0.0±0.0 | 0.0±0.0 | 0.0±0.0 | 0.0±0.0 | 0.0±0.0 | 0.0±0.0 |
| 36 | 18.217 | Linoleic acid, methyl ester |  | 0.1±0.1 | 0.0±0.0 | 0.01±0.0 | 0.0±0.0 | 0.0±0.0 | 0.0±0.0 | 0.0±0.0 | 0.0±0.0 |
| 37 | 18.295 | Oleic acid, methyl ester |  | 0.1±0.1 | 0.0±0.0 | 0.04±0.1 | 0.0±0.0 | 0.0±0.0 | 0.0±0.0 | 0.0±0.0 | 0.02±0.0 |
| **Total fatty acid/esters (%)** | | | | **31.3±2.4** | **32.0±4.4** | **37.8±5.8** | **32.4±3.2** | **37.2±4.2** | **32.4±6.9** | **42.1±4.6** | **35.8±5.7** |
| 38 | 10.997 | 6-Methoxy-3-methylbenzofuran | **Furan** | 0.2±0.1 | 0.1±0.0 | 0.1±0.0.1 | 0.1±0.0 | 0.1±0.1 | 0.1±0.1 | 0.2±0.1 | 0.1±0.0 |
| 39 | 6.708 | Acetophenone | **Ketone** | 1.1±0.2 | 1.4±0.3 | 2.2±0.5 | 2.2±0.7 | 2.3±0.7 | 3.1±1.4 | 2.3±0.9 | 1.9±0.4 |
| 40 | 6.268 | *β*-Phellandrene | **Monoterpene hydrocarbon** | 0.6±0.2 | 0.0±0.0 | 0.0±0.0 | 0.1±0.0 | 0.1±0.0 | 0.0±0.0 | 0.0±0.0 | 0.0±0.0 |
| 41 | 7.504 | D-Limonene* |  | 1.5±0.6 | 1.1±0.2 | 2.4±0.8 | 1.6±0.8 | 2.3±0.6 | 3.0±1.1 | 1.9±0.8 | 1.3±0.5 |
| 42 | 7.694 | 3-Carene |  | 0.4±0.1 | 0.3±0.1 | 0.4±0.1 | 0.3±0.1 | 0.4±0.1 | 0.3±0.1 | 0.4±0.1 | 0.2±0.1 |
| 43 | 7.849 | γ-Terpinene |  | 0.5±0.1 | 0.0±0.0 | 0.1±0.0.0 | 0.1±0.0 | 0.1±0.0 | 0.1±0.0 | 0.1±0.0 | 0.1±0.0 |
| **Total monoterpene hydrocarbons (%)** | | | | **2.9±0.9** | **1.4±0.3** | **3.0±1.0** | **2.1±0.9** | **2.8±0.8** | **3.4±1.2** | **2.3±0.9** | **1.6±0.6** |
| 44 | 9.354 | Estragole* | **Phenol/Ether** | 3.5±0.2 | 1.2±0.2 | 0.5±0.1 | 1.8±0.2 | 0.6±0.2 | 0.5±0.2 | 0.7±0.1 | 1.6±0.2 |
| 45 | 9.69 | Thymol methyl ether |  | 0.1±0.0 | 0.1±0.0 | 0.1±0.0 | 0.1±0.0 | 0.1±0.0 | 0.1±0.0 | 0.1±0.0 | 0.1±0.0 |
| 46 | 9.895 | Unknown |  | 1.8±0.4 | 0.9±0.1 | 0.4±0.1 | 1.1±0.7 | 0.4±0.1 | 0.2±0.0 | 0.5±0.1 | 0.8±0.2 |
| 47 | 10.274 | Anethole* |  | 17.3±0.7 | 20.4±0.0 | 9.1±7.9 | 23.3±0.5 | 15.9±2.0 | 12.8±3.1 | 10.0±8.6 | 18.2±5.4 |
| 48 | 10.882 | Eugenol* |  | 0.1±0.0 | 0.1±0.0 | 0.0±0.0 | 0.0±0.0 | 0.0±0.0 | 0.0±0.0 | 0.1±0.0 | 0.1±0.0 |
| 49 | 11.525 | 2-Allyl-1,4-dimethoxybenzene |  | 0.03±0.0 | 0.03±0.0 | 0.02±0.0 | 0.0±0.0 | 0.0±0.0 | 0.0±0.0 | 0.0±0.0 | 0.0±0.0 |
| 50 | 12.084 | Methylisoeugenol |  | 0.5±0.1 | 0.4±0.1 | 0.2±0.0 | 0.2±0.1 | 0.1±0.0 | 0.1±0.0 | 0.1±0.0 | 0.3±0.1 |
| 51 | 12.462 | Myristicin* |  | 1.5±0.2 | 0.1±0.0 | 0.0±0.0 | 0.2±0.1 | 0.0±0.0 | 0.1±0.0 | 0.0±0.0 | 0.1±0.0 |
| 52 | 15.758 | Ethoxy-4-methoxybenzaldehyde |  | 0.03±0.03 | 0.0±0.0 | 0.0±0.0 | 0.0±0.0 | 0.0±0.0 | 0.0±0.0 | 0.0±0.0 | 0.01±0.0 |
|  |  |  |  | **Barhi** | **Om Eldehn** | **Rothana** | **Lolo** | **Nabout Seif** | **Khalas** | **Farsi** | **Breem** |
| **Total phenol/ethers (%)** | | | | **24.9±1.6** | **23.1±0.4** | **10.3±8.0** | **26.8±1.6** | **17.1±2.3** | **13.8±3.4** | **11.5±8.9** | **21.2±6.0** |
| 53 | 10.737 | *δ*-EIemene | **Sesquiterpene hydrocarbon** | 0.5±0.0 | 0.5±0.1 | 0.2±0.0 | 0.2±0.0 | 0.1±0.0 | 0.1±0.0 | 0.1±0.0 | 0.2±0.1 |
| 54 | 10.925 | *α*-Longipinene |  | 0.2±0.2 | 0.1±0.00.0 | 0.1±0.0 | 0.1±0.0 | 0.0±0.0 | 0.0±0.0 | 0.1±0.0 | 0.1±0.0 |
| 55 | 11.239 | *β*-Elemene |  | 0.2±0.0 | 0.2±0.0 | 0.1±0.0 | 0.1±0.0 | 0.0±0.0 | 0.0±0.0 | 0.0±0.0 | 0.1±0.0 |
| 56 | 11.554 | *α*-Bisabolene |  | 0.05±0.0 | 0.03±0.0 | 0.03±0.0 | 0.02±0.0 | 0.01±0.0 | 0.01±0.0 | 0.02±0.0 | 0.02±0.0 |
| 57 | 11.609 | *cis*-*α*-Bergamotene |  | 0.3±0.0 | 0.2±0.0 | 0.1±0.0 | 0.1±0.0 | 0.1±0.0 | 0.0±0.0 | 0.1±0.0 | 0.1±0.0 |
| 58 | 11.718 | *cis*-*β*-Farnesene |  | 0.2±0.0 | 0.2±0.0 | 0.1±0.0 | 0.1±0.0 | 0.0±0.0 | 0.0±0.0 | 0.1±0.0 | 0.1±0.0 |
| 59 | 11.857 | *α*-Himachalene |  | 0.7±0.0 | 0.6±0.1 | 0.3±0.0 | 0.3±0.1 | 0.2±0.0 | 0.1±0.0 | 0.2±0.0 | 0.4±0.1 |
| 60 | 11.935 | *β*-Vatirenene |  | 0.1±0.0 | 0.0±0.0 | 0.0±0.0 | 0.0±0.0 | 0.0±0.0 | 0.0±0.0 | 0.0±0.0 | 0.0±0.0 |
| 61 | 12.004 | Eudesma-2,4,11-triene |  | 0.6±0.2 | 0.6±0.0 | 0.3±0.0 | 0.2±0.1 | 0.1±0.0 | 0.1±0.0 | 0.1±0.0 | 0.3±0.1 |
| 62 | 12.04 | *α*-Curcumene |  | 1.9±0.1 | 1.7±0.3 | 0.9±0.1 | 1.0±0.3 | 0.5±0.1 | 0.3±0.0 | 0.7±0.1 | 1.3±0.3 |
| 63 | 12.131 | *α*-Guaiene |  | 3.1±0.1 | 2.9±0.1 | 2.0±0.2 | 2.1±0.2 | 1.4±0.2 | 0.9±0.2 | 1.6±0.1 | 2.5±0.2 |
| 64 | 12.3 | *β*-Bisabolene |  | 1.0±0.1 | 0.9±0.1 | 0.4±0.0 | 0.5±0.2 | 0.2±0.1 | 0.1±0.1 | 0.3±0.0 | 0.6±0.1 |
| 65 | 12.648 | *γ*-Dehydro-ar-himachalene |  | 0.2±0.0 | 0.1±0.0 | 0.1±0.0 | 0.1±0.0 | 0.0±0.0 | 0.0±0.0 | 0.0±0.0 | 0.1±0.0 |
| **Total sesquiterpene hydrocarbons (%)** | | | | **9.0±0.5** | **8.0±0.9** | **4.5±0.5** | **4.8±1.2** | **2.8±0.6** | **1.8±0.5** | **3.3±0.3** | **5.8±0.9** |

* Compounds with asterisk are confirmed by comparison with standards

**Table S4**: Mineral content in different cvs. of *Phoenix dactylifera* L. seeds expressed as mean (ppm) mean ± SD), n = 3

| **Cultivar name** | **Ca** | **Mg** | **P** | **Fe** | **Cu** | **Se** | **Mn** | **B** |
| --- | --- | --- | --- | --- | --- | --- | --- | --- |
| **Barhi** | 2566.33 ± 10.12 | 1393.00 ± 1.00 | 1665 ± 870.39 | 4199.00 ± 26.85 | 218.70 ± 1.13 | 42.70 ± 2.72 | 186.03 ± 6.28 | 6.73 ± 0.20 |
| **Omeldehn** | 891.33 ± 6.43 | 850.00 ± 6.00 | 1075.67 ± 7.57 | 1499.67 ± 11.02 | 45.85 ± 0.90 | 9.40 ± 1.82 | 106.14 ± 2.95 | 2.81 ± 0.10 |
| **Rothana** | 685.33 ± 6.81 | 710.00 ± 5.57 | 962.67 ± 14.22 | 1037.00 ± 6.24 | 45.62 ± 0.57 | 11.20 ± 0.41 | 74.11 ± 2.66 | 0.82 ± 0.05 |
| **Lolo** | 813.33 ± 6.51 | 729.33 ± 2.52 | 1064.67 ± 4.51 | 1494.00 ± 14.00 | 57.00 ± 0.41 | 44.12 ± 1.99 | 104.32 ± 0.36 | 3.91 ± 0.25 |
| **Nabout Seif** | 550.00 ± 7.21 | 779.33 ± 7.77 | 1139.00 ± 90.52 | 847.33 ± 2.52 | 46.35 ± 1.52 | 62.64 ± 1.52 | 88.49 ± 4.67 | 4.50 ± 0.12 |
| **Zamli** | 2902.33 ± 8.50 | 1469.00 ± 8.66 | 1762.00 ± 23.07 | 5771.00 ± 17.44 | 73.94 ± 1.15 | 14.38 ± 1.48 | 184.27 ± 4.58 | 6.10 ± 0.14 |
| **Khalas** | 173.00 ± 2.65 | 701.67 ± 8.50 | 1024.67 ± 15.70 | 247.33 ± 10.07 | 37.93 ± 1.51 | 53.83 ± 1.37 | 100.58 ± 2.91 | 2.21 ± 0.15 |
| **Farsi** | 341.67 ± 2.31 | 412.67 ± 2.08 | 674.67 ± 1.15 | 454.67 ± 0.58 | 20.49 ± 0.30 | 33.93 ± 1.13 | 52.67 ± 2.04 | 0.48 ± 0.14 |
| **Hasawi** | 614.67 ± 5.77 | 689.67 ± 9.24 | 977.00 ± 5.00 | 415.67 ± 3.79 | 42.64 ± 0.94 | 104.05 ± 4.43 | 75.16 ± 4.38 | 0.69 ± 0.20 |
| **Zaghloul** | 663.67 ± 3.51 | 879.67 ± 10.02 | 1130.00 ± 11.79 | 839.33 ± 6.03 | 50.85 ± 0.05 | 3.82 ± 0.77 | 80.51 ± 2.54 | 0.50 ± 0.02 |
| **Aref** | 539.33 ± 0.58 | 705.33 ± 3.51 | 1088.00 ± 367.19 | 1388.33 ± 6.51 | 43.44 ± 0.67 | 26.31 ± 0.96 | 96.19 ± 0.82 | 1.14 ± 0.19 |
| **Breem** | 860.33 ± 1.53 | 728.67 ± 16.74 | 1061.33 ± 4.51 | 1377.33 ± 6.43 | 47.43 ± 0.43 | - | 116.64 ± 2.29 | 1.35 ± 0.31 |

# **Supplementary Figures**


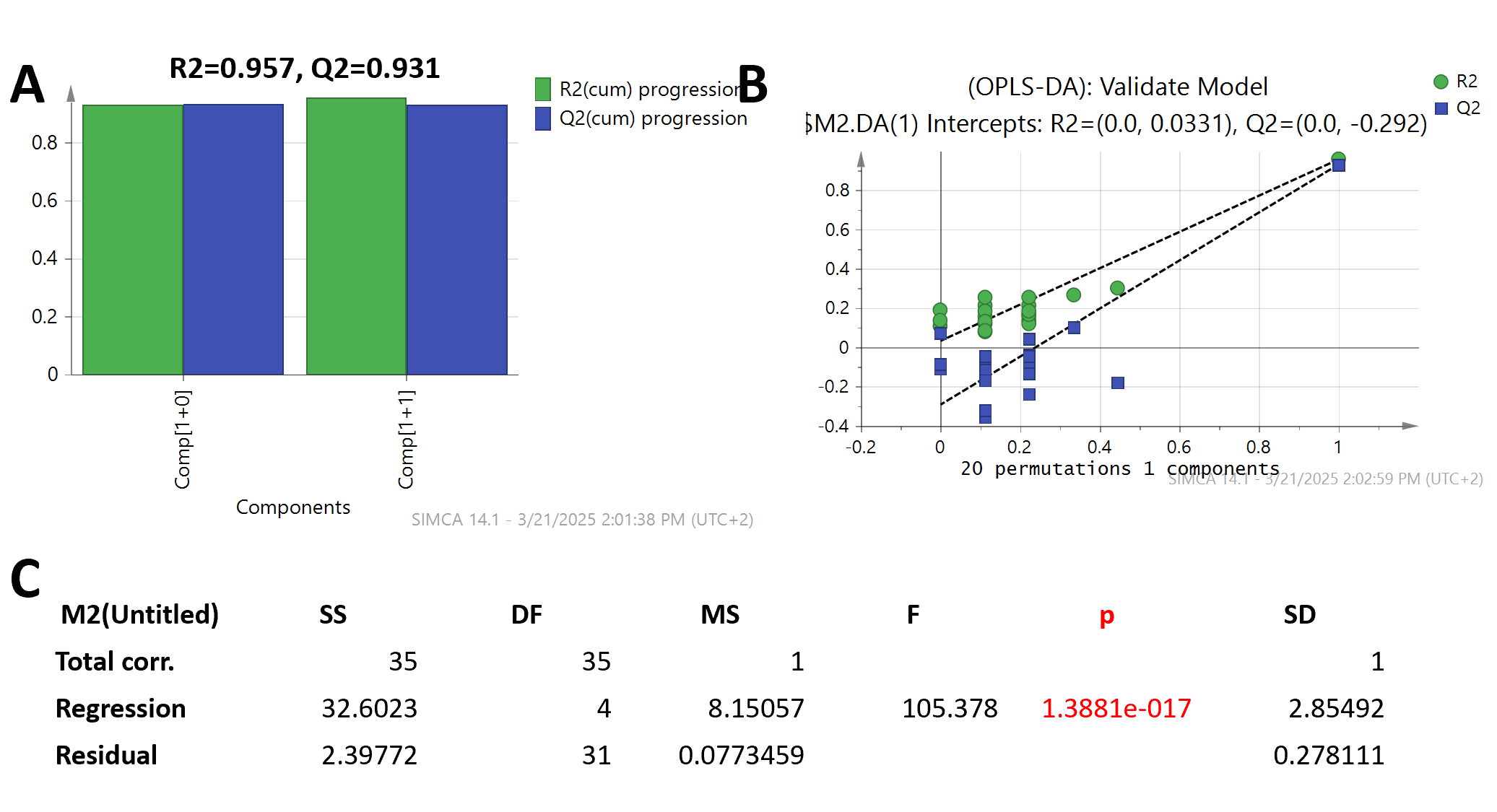


**Figure S1:** Optimization and validation parameters for supervised orthogonal partial least squares discriminant analysis (OPLS-DA) modelling of post silylation GC-MS dataset of group 1 against group 2 varieties as appeared in HCA model **Figure 6D**. **A)** The diagnostic metrics R^2^Y and Q^2^ as function of number of principal components. **B)** Permutation test (n= 20) that showed negative Q^2^ intercept value. **C)** CVANOVA to assess model statistical significance

**Figure S2:** Optimization and validation parameters for supervised orthogonal partial least squares discriminant analysis (OPLS-DA) modelling of Barhi VOCs profile against Group 2 all other varieties. **A)** The diagnostic metrics R^2^Y and Q^2^ as function of number of principal components. **B)** Permutation test (n= 100) that showed negative Q^2^ intercept value. **C)** CVANOVA to assess model statistical significance
